# Vascular Effects of the Polyphenolic Nutraceutical Supplement Taurisolo^®^: Focus on the Protection of the Endothelial Function

**DOI:** 10.3390/nu13051540

**Published:** 2021-05-02

**Authors:** Alma Martelli, Lorenzo Flori, Era Gorica, Eugenia Piragine, Anella Saviano, Giuseppe Annunziata, Matteo Nicola Dario Di Minno, Roberto Ciampaglia, Ilenia Calcaterra, Francesco Maione, Gian Carlo Tenore, Ettore Novellino, Vincenzo Calderone

**Affiliations:** 1 Department of Pharmacy, University of Pisa, Via Bonanno 6, 56126 Pisa, Italy; lorenzo.flori@phd.unipi.it (L.F.); era.gorica@phd.unipi.it (E.G.); eugenia.piragine@farm.unipi.it (E.P.); vincenzo.calderone@unipi.it (V.C.); 2 Interdepartmental Research Center “Nutrafood: Nutraceutica e Alimentazione per la Salute”, University of Pisa, 56126 Pisa, Italy; 3 Interdepartmental Research Center “Biology and Pathology of Ageing”, University of Pisa, 56126 Pisa, Italy; 4 ImmunoPharmaLab, Department of Pharmacy, School of Medicine and Surgery, University of Naples Federico II, Via Domenico Montesano 49, 80131 Naples, Italy; anella.saviano@unina.it (A.S.); francesco.maione@unina.it (F.M.); 5 Department of Pharmacy, University of Naples Federico II, Via Domenico Montesano 49, 80131 Naples, Italy; giuseppe.annunziata@unina.it (G.A.); roberto.ciampaglia@unina.it (R.C.); giancarlo.tenore@unina.it (G.C.T.); ettore.novellino@unina.it (E.N.); 6 Department of Translational Medical Sciences, University of Naples Federico II, Via Sergio Pansini 5, 80131 Naples, Italy; dario.diminno@hotmail.it; 7 Department of Clinic Medicine and Surgery, University of Naples Federico II, Via Sergio Pansini 5, 80131 Naples, Italy; ileniacalcaterra@hotmail.it

**Keywords:** endothelial dysfunction, vascular protection, Taurisolo^®^, polyphenolic extract, hypertension, sirtuins, AMPK, nitric oxide, nutraceutical supplement

## Abstract

Preservation of vascular endothelium integrity and functionality represents an unmet medical need. Indeed, endothelial dysfunction leads to decreased nitric oxide biosynthesis, which is prodromic of hypertension and hypercoagulability. In this panorama, the nutraceutical supplement Taurisolo^®^, a polyphenolic extract from Aglianico cultivar grape, rich in catechin and procyanidins, was evaluated as a vasoprotective, vasorelaxing, anti-hypertensive and anti-coagulant agent in: cell lines, isolated vessels, in vivo models of chronic hypertension and hypercoagulability, and in clinical tests of endothelial reactivity. Taurisolo^®^ demonstrated to fully protect vascular cell viability from oxidative stimulus at 100 µg/mL and evoke vasorelaxing effects (Emax = 80.6% ± 1.9 and pEC50 = 1.19 ± 0.03) by activation of the Sirtuins-AMPK-pathway. Moreover, Taurisolo^®^, chronically administered at 20 mg/Kg/die in in vivo experiments, inhibited the onset of cardiac hypertrophy (heart weight/rat weight = 3.96 ± 0.09 vs. 4.30 ± 0.03), hypercoagulability (decrease of fibrinogen vs. control: *p* < 0.01) and hypertension (mean of Psys: 200 ± 2 vs. control 234 ± 2 mmHg) and improved endothelial function (Emax = 88.9% ± 1.5 vs. control 59.6% ± 3.6; flow-mediated dilation in healthy volunteers after 400 mg twice daily for 8 weeks vs. baseline: *p* = 0.019). In conclusion, Taurisolo^®^ preserves the vascular function against ox-inflamm-ageing process and the consequent cardiovascular accidents.

## 1. Introduction

Protection of the vascular system against endothelial degenerative processes and vascular wall injury represents a hot topic in cardiovascular research, because of the lack of specific/approved therapeutic approaches or preventive strategies. Thus, this pathological condition could be defined as an “unmet medical need”. The vascular system impairment often starts with oxidative and inflammatory processes which trigger the endothelial dysfunction and then spread to vascular smooth muscle. This process often remains silent for a long time and derives in part from the progressive ageing of vascular tissues, in part from a fat-rich diet and in part from the presence of metabolic diseases such as metabolic syndrome, diabetes, obesity and dyslipidemias. Although apparently heterogeneous, all these conditions share both systemic inflammatory status and inadequate control of redox homeostasis. Endothelial dysfunction represents the earliest stage of the atherosclerotic process and is a recognized trigger of vascular events [1]. In addition, impairment of endothelial function is widespread in “supposedly healthy” people, starting from about 40 years of age. If ignored, it often progresses towards symptomatic cardiovascular diseases such as hypertension, stroke, myocardial infarct, erectile dysfunction and cognitive impairment [2]. In this panorama, a nutraceutical approach characterized by the ability to prevent endothelial dysfunction and support the physiological vascular function seems to be the most appropriate strategy. The grape pomace polyphenolic extract Taurisolo^®^ (TAU), microencapsulated with maltodextrins, is a nutraceutical product obtained from *Aglianico* cultivar grape, rich in resveratrol but particularly in catechin and procyanidins. Indeed, Taurisolo^®^, is administered in an acid-resistant formulation that effectively protect bioactive compounds against a partial degradation caused by gastrointestinal (GI) digestion. Moreover, microencapsulation of Taurisolo^®^ polyphenols improves their bioavailability, resulting in a rapid absorption across the intestinal mucosa [3]. This is confirmed by pharmacokinetic studies reporting the serum peak of Taurisolo^®^ polyphenols at 60 min after oral administration [4]. Finally, previous pilot clinical trials demonstrated its ability to reduce the levels of trimethylamine-N-oxide (TMAO), which represents a recognized cardiovascular risk factor, especially for atherogenic process [4,5]. More recently, it has been demonstrated that TMAO, which is generated by gut microbiota, causes vascular inflammation and endothelial injury, triggering atherosclerotic plaque formation [6].

In this experimental study, Taurisolo^®^ underwent a pharmacological characterization in order to evaluate its ability to protect the vascular wall against oxidative stimuli, to induce vasodilation, and when chronically administered, to inhibit the onset of endothelial dysfunction, cardiac hypertrophy, hypercoagulability and hypertension, also investigating its potential mechanism of action at the vascular level.

## 2. Materials and Methods

### 2.1. Taurisolo^®^ Supplement

Taurisolo^®^ is a nutraceutical supplement consisting of a polyphenol extract obtained from *Aglianico* cultivar grape, collected during the autumn 2018 harvest. Firstly, the Department of Pharmacy, University of Naples Federico II (Naples, Italy), provided the supplement formulation, then the large-scale production was accomplished by MB-Med Company (Turin, Italy). For the Taurisolo^®^ production and composition see Supplemental Materials and previous work [7].

### 2.2. Cell Cultures

Human aortic smooth muscle cells, HASMCs (Life Technologies, Carlsbad, CA, USA) and Human umbilical vein endothelial cells, HUVECs (Life Technologies, Carlsbad, CA, USA) were cultured and seeded as reported in Supplemental Materials.

#### 2.2.1. Evaluation of the Cell Viability Preservation against H_2_O_2_-Induced Cell Damage in HASMCs and HUVECs

After 24 h, to allow cell attachment, the medium was replaced by fresh culture medium, and cells were treated with TAU 10, 30 and 100 μg/mL or vehicle for 1 h. This concentration range was selected on the basis of preliminary set-up experiments (data not shown). After 1 h, cells were challenged with the oxidant stimulus, represented by H_2_O_2_ (200 μM for HASMCs and 100 μM for HUVECs). At the end of the 2 h of H_2_O_2_ incubation, the cell viability was assessed using an aqueous solution of the cell proliferation reagent WST-1 (Roche, Basilea, Switzerland), (see Supplemental Materials). 

#### 2.2.2. Evaluation of Cell Viability Preservation against H_2_O_2_-Induced Cell Damage in HASMCs and HUVECs in the Presence of Sirtuins and AMPK Inhibitors 

After 24 h, to allow cell attachment, the medium was replaced and both the cell lines were incubated for 1 h with Sirtinol (Sirt, a sirtuins inhibitor; Tocris Bio-Techne, Minneapolis, MN, USA) or Compound C (CC, also known as dorsomorphin, an AMPK inhibitor; Merck KGaA, Darmstadt, Germany) at the concentration of 10 μM, or both, or their vehicle. After 1 h of incubation, Taurisolo^®^ 100 μg/mL or vehicle (culture medium) was added and incubated for 1 h. After 1 h, the cells were incubated for 2 h with the pro-oxidant agent represented by H_2_O_2_ (200 μM for HASMCs and 100 μM for HUVECs). Cell viability was assessed using an aqueous solution of the cell proliferation reagent WST-1 (Roche, Basilea, Switzerland), (see Supplemental Materials). 

#### 2.2.3. Measurement of Intracellular H_2_O_2_-Induced ROS Production in HUVECs and HASMCs in the Presence of Sirtuins and AMPK Inhibitors

Cells were treated as reported in Section 2.2.2 and, at the end of the treatment, intracellular levels of reactive oxygen species (ROS) were measured (see Supplemental Materials). 

#### 2.2.4. Cell Experiments Data Analysis

The experiments were carried out in triplicate and repeated at least three times (n = 9), and the values obtained were expressed as a mean ± standard error (SEM). The data were analyzed by using the ANOVA one way test followed by Bonferroni’s Multiple Comparison *post hoc* test; a level of *p* < 0.05 was considered to be a statistical significance limit (* *p*< 0.05; ** *p*< 0.01; *** *p*< 0.001).

### 2.3. Animals Protocols and Ethical Statements 

All experimental procedures were carried out according to the Code of Ethics of the World Medical Association (Declaration of Helsinki, EU, Directive 2010/63/EU for animal experiments) and following the guidelines of the European Community Council Directive 86-609. The experiments were authorized by the Ethical Committee of the University of Pisa (Protocol number: 0037321/2013) and by the Italian Ministry of Health (authorization number 487/2020-PR). Animal studies were carried out in compliance with the ARRIVE guidelines and the Basel Declaration including the 3Rs concept [8,9]. All procedures were carried out to minimize the number of animals used and their suffering.

#### 2.3.1. Evaluation of the Vasorelaxing Effect of Taurisolo^®^ on Rat Aorta Rings in the Presence and Absence of Endothelium

Three-month-old male Wistar rats (280–350 g) housed in cages with free access to food and water, were sacrificed with an overdose of Sodium Thiopental 100 mg/kg (MSD Animal Health, Milan, Italy), and thoracic descending aorta segment was excised and mounted as described in Supplemental Materials and in previous works [10,11].

At the end of the stabilization period, the aortic rings were challenged again with KCl 25 mM to induce vasoconstriction and, once reached a stable plateau, increasing cumulative concentrations of Taurisolo^®^ (0.003; 0.01; 0.03; 0.1; 0.3; 1; 3 mg/mL) were administered in aortic rings with and without endothelium. To verify the involvement of endogenous nitric oxide (NO) in the endothelium-dependent vasorelaxation induced by Taurisolo^®^, the aortic rings with intact endothelium were pre-incubated with the inhibitor of NO biosynthesis N(ω)-nitro-l-arginine methyl ester (L-NAME) 100 μM (Merck KGaA, Darmstadt, Germany) for 20 min, before the administration of increasing cumulative concentrations of Taurisolo^®^. The vasorelaxing effect promoted by Taurisolo^®^ was expressed as described in Supplemental Materials and in previous works [12,13].

#### 2.3.2. Evaluation of the Involvement of Sirtuins and AMPK Pathways in the Vasorelaxing Activity of Taurisolo^®^

The potential mechanisms of action, accounting for the vasorelaxing effect exhibited by Taurisolo^®^ on the endothelium-intact aortic rings, were investigated through the pre-incubation of the vessels, for 1 h, with the sirtuins inhibitor Sirtinol (100 μM) or the AMPK inhibitor Compound C (100 μM) or both at the concentration of 100 μM. Therefore, after reaching a stable plateau obtained by administration of KCl 25 mM, increasing concentrations of Taurisolo^®^ (0.003; 0.01; 0.03; 0.1; 0.3; 1; 3 mg/mL) were administered to each aortic ring. The vasorelaxing effect, observed after the administration of each concentration of Taurisolo^®^, was expressed as described in Supplemental Materials.

#### 2.3.3. Evaluation of the Efficacy of Taurisolo^®^ to Restrain Noradrenaline (NA)-Induced Vasoconstriction 

Three different concentrations of Taurisolo^®^ (10, 30, 100 μg/mL) or the corresponding vehicle (deionized water) were pre-incubated for 20 min on different isolated endothelium-intact aortic rings. Then, increasing concentrations of NA (Merck KGaA, Darmstadt, Germany) (10^−9^ M; 3 × 10^−9^ M; 10^−8^ M; 3 × 10^−8^ M; 10^−7^ M; 3 × 10^−7^ M; 10^−6^ M) were added in each aortic ring. Finally, after the NA-cumulative concentration–response curve, the aortic rings were washed and, after a stabilization period of 20 min, KCl 60 mM was administered to obtain a maximal contraction (100%). The vasocontracting effect of each NA concentration was expressed as described in Supplemental Materials and in a previous work [13].

#### 2.3.4. In Vitro Experiments Statistical Analysis

Experimental data were analyzed by a computer fitting procedure (software: GraphPad Prism 6.0). The experiments were conducted in triplicate and repeated at least three times (n = 9). The statistical significance was obtained by the Two-Way ANOVA statistical analysis followed by Bonferroni post-test. Values were considered statistically different when *p* < 0.05. 

### 2.4. Anti-Hypertensive Effects of Taurisolo^®^ in Spontaneously Hypertensive Rats (SHRs) In Vivo Model

Twenty SHRs (Charles River Laboratories, Calco, Italy) of 6 weeks of age were randomized into 4 groups (5 animals for each group). The 4 groups daily received (*per os*, dissolved in the drinking water) 4 different treatments for 4 weeks: (1) Tap water (control group), (2) Taurisolo^®^ 10 mg/kg, (3) Taurisolo^®^ 20 mg/kg, and (4) Captopril 20 mg/kg (Merck KGaA, Darmstadt, Germany) used as reference anti-hypertensive drug. Systolic blood pressure and other parameters were recorded as reported in Supplemental Materials and in previous studies [14,15]. The statistical significance was obtained by the Two-Way ANOVA statistical analysis followed by Bonferroni post-test. Statistical significance was set at *p* < 0.05.

#### 2.4.1. Evaluation of Taurisolo^®^ Protection against Endothelial Dysfunction in SHRs 

At the end of the chronic in vivo treatment, the SHRs thoracic aortas were removed, and rings were set up as previously described, to assess the possible protection of Taurisolo^®^ against endothelial dysfunction by the widely recognized “Acetylcholine (Ach) vasorelaxing test” as described in Supplemental Materials and in a previous work [16]. 

The statistical significance was obtained by the Two-Way ANOVA statistical analysis followed by Bonferroni post-test. Statistical significance was set at *p* < 0.05.

#### 2.4.2. Effect of Taurisolo^®^ on Glycemic and Lipid Parameters in SHRs

At the end of the 4-week of chronic in vivo treatment, the lipid (total cholesterol, TC; high-density lipoprotein, HDL; low-density lipoprotein, LDL; triglycerides, TG) and glycemic profiles were also investigated as reported in Supplemental Materials. 

#### 2.4.3. Preventive Effects of Taurisolo^®^ against Cardiac Hypertrophy in SHRs

At the end of the chronic treatment, the heart of each animal was removed, washed, dried, dissected and finally weighed to assess the preventive effects of Taurisolo^®^ against the development of cardiac hypertrophy in SHRs as described in Supplemental Materials and in a previous work [15]. Significance was obtained with One-Way ANOVA statistical analysis followed by Bonferroni post-test. Data were considered statistically different when *p* < 0.05.

### 2.5. Measurement of Coagulation Factors and Fibrinogen 

Mice were randomly separated into four experimental groups of six animals each, balancing body weight variation across groups. Taurisolo^®^ at a dose of 1, 10 and 20 mg/kg and distilled water (TAU vehicle; Ctrl group) were administered orally (p.o.; 200 μL/mouse) for 4 weeks. Thereafter, haematological investigations of coagulation factors, including prothrombin time (PT; expressed as seconds), partial thromboplastin time (PTT; expressed as seconds) and fibrinogen (expressed as mg/dL) were performed as described in Supplemental Materials and in previous works [17,18]. 

#### Clot Retraction Assay

For the clot retraction assay, we adopted the protocol proposed by Law and coll. with slight modifications (see Supplemental Materials) [19,20].

The data and statistical analysis in 2.5 experimental procedures complied with the international recommendations on experimental design and analysis in pharmacology [21] and data sharing and presentation in preclinical pharmacology [22,23]. The results obtained were expressed as the mean ± SD. Statistical analyses were performed by using One-Way ANOVA followed by Bonferroni’s for multiple comparisons. GraphPad Prism 8.0 software (San Diego, CA, USA) was used for analysis. Data were considered statistically significant when a value of *p* ≤ 0.05 was achieved.

### 2.6. Human Clinical Studies on the Effects of Taurisolo^®^ on Endothelial Function

#### 2.6.1. Study Population and Protocol

In the frame of a randomized, double-blind, placebo-controlled, parallel-arm clinical trial, healthy volunteers were recruited according to criteria reported in Supplemental Materials. 

Overall study duration was 12 weeks: 2-week run-in period (without Taurisolo^®^ supplementation); 8-week intervention period with Taurisolo^®^ (400 mg twice daily) or placebo (400 mg maltodextrin twice daily) and 2-week follow-up period. The examinations were performed in an outpatient setting. Clinical examinations and blood sampling were performed after 12 h of fasting at weeks 2 and 10 according to the procedures described in Supplemental Materials. 

Study participants were randomized into two treatment arms: active group (receiving Taurisolo^®^ treatment) and placebo group (receiving maltodextrins). This study was conducted both in acute and chronic. For the acute study, 800 mg of Taurisolo^®^ or maltodextrins were administered to each study participant randomized into the relative intervention group. For the chronic study, subjects in the active group were administered with 400 mg Taurisolo^®^ twice daily, while subjects in the placebo group were administered with equivalent dose of maltodextrins.

A total of 30 subjects were enrolled. If a patient dropped out before the intervention period, he/she was replaced by the next eligible patient enrolled at the same center. Patients, clinicians, core laboratories, and trial staff (data analysts, statisticians) were blind to treatment allocation. 

Before giving their written consent, all study participants received oral and written information about the study. The protocol, volunteers’ letters of intent, and a synoptic paper regarding the study were sent to the Scientific Ethics Committee of AO Rummo Hospital (Benevento, Italy) which approved the study (protocol 123,512 of 18/06/2018, carried out in accordance with the Helsinki declaration of 1964, as revised in 2000). The participants were asked to make records in an intake-checking table for the intervention study and side effects in daily reports.

Additional information concerning the study protocol, including study procedures and statistical analyses, are detailed in the Supplemental Materials.

#### 2.6.2. Blood Parameter Analyses

All biochemical analyses, including fasting plasma glucose, total cholesterol, fasting plasma triglycerides, HDL and LDL cholesterol were performed with a fully automated analyzer (Sphera, Edif s.r.l., Rome, Italy).

Serum levels of oxidized low-density lipoproteins (oxLDL) and reactive oxygen metabolites (D-ROMs), as oxidative stress-related biomarkers, were assessed with an automated analyzed (Free Carpe Diem, Diacron International, Grosseto, Italy) using relative commercial kits (Diacron International) according to the manufacture’s instruction, as previously reported [5,24]. Methods are described in detail in Supplemental Materials.

#### 2.6.3. Brachial Artery Flow-Mediated Dilation (FMD) and Reactive Hyperemia Index (RHI)

Participants had to abstain from tobacco, caffeine, and alcohol for at least 12 h before the evaluation. All the procedures were performed in a temperature-controlled room (23 °C), after overnight fasting and after ≥10 min of rest in supine position (a small head pillow was accepted). Brachial artery FMD and RHI were assessed at baseline (before Taurisolo^®^ or maltodextrin administration), in the acute phase (1 h after 800 mg Taurisolo^®^ or maltodextrin administration) and at t8 (after 8-weeks of supplementation with 400 mg Taurisolo^®^ twice daily or 400 mg maltodextrin twice daily). All assessments were performed by the same expert operator, blinded for ongoing treatment. FMD and RHI were measured by ultrasound imaging, as described in the guidelines of the International Brachial Artery Reactivity Task Force [1].

FMD and RHI of the brachial artery were evaluated according to a standardized ultrasound protocol [25] using an automatic edge detection software (Cardiovascular Suite^®^, FMD studio, QUIPU Srl, Pisa, Italy) as described in Supplemental Materials and in a previous work [26].

## 3. Results

### 3.1. Evaluation of the Protective Effect Induced by Taurisolo^®^ on HUVEC and HASMC Cell Viability, against the H_2_O_2_-Induced Oxidative Damage

**Figure 1:**
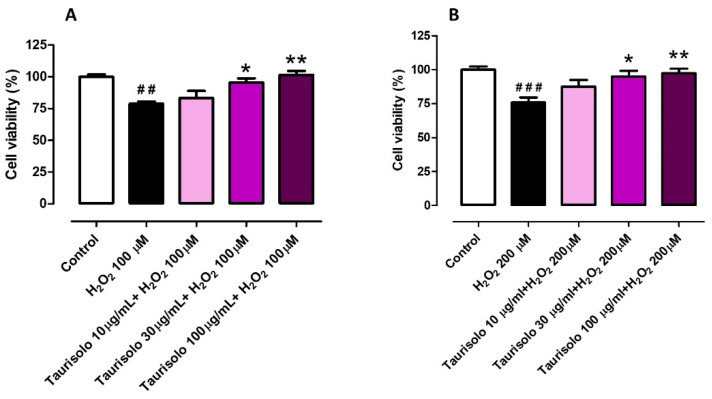
Evaluation of the protective effect induced by Taurisolo^®^ on HUVECs (**A**) and HASMCs (**B**) cell viability, against the H_2_O_2_-induced oxidative damage. The colored histograms indicate the % of cell viability of treated cells vs. the value of cell viability in cells treated only with vehicle (Control, white histogram). All the experiments were repeated for a minimum of three times and each one was carried out in triplicate (n = 9). The symbols indicate the level of statistical significance calculated by ANOVA one-way, followed by Bonferroni post-test: ^#^ indicates the significance vs. Control, * indicates significance vs. cells treated with H_2_O_2_ (^##^ *p* < 0.01, ^###^ *p* < 0.001; * *p* < 0.05, ** *p* < 0.01).

Taurisolo^®^ was tested to evaluate its ability to protect both HUVECs and HASMCs against an oxidative stimulus obtained by the administration of H_2_O_2_. HUVECs were challenged with H_2_O_2_ 100 μM and HASMCs with H_2_O_2_ 200 μM, according to preliminary experiments carried out to establish their different sensitivity to the oxidative stimulus (data not shown). The administration of H_2_O_2_ on HUVECs induced a decrease of cell viability by about 21% (%cell viability vs. control: 78.9 ± 1.5). This reduction was prevented, in a concentration-dependent manner, by the pre-incubation of three different concentrations of Taurisolo^®^ (%cell viability vs. control for 10 µg/mL: 83.3 ± 5.6, for 30 µg/mL: 95.6 ± 3.2 and for 100 µg/mL: 101.6 ± 3.2) (Figure 1A). On the other hand, the administration of H_2_O_2_ on HASMCs induced a decrease of cell viability by about 24% (%cell viability vs. control: 75.8 ± 3.7) and also in HASMCs, the pre-incubation of the three different concentrations of Taurisolo^®^ induced a concentration-dependent trend of protection (%cell viability vs. control for 10 µg/mL: 87.5 ± 4.9, for 30 µg/mL: 94.9 ± 4.2 and for 100 µg/mL: 97.2 ± 3.4) (Figure 1B).

### 3.2. Investigation of the Potential Mechanisms of Action Involved in the Protective Effect Induced by Taurisolo^®^ on HUVEC and HASMC Cell Viability, against the H_2_O_2_-Induced Oxidative Damage. Evaluation of Sirtuins and AMPK Pathways

**Figure 2:**
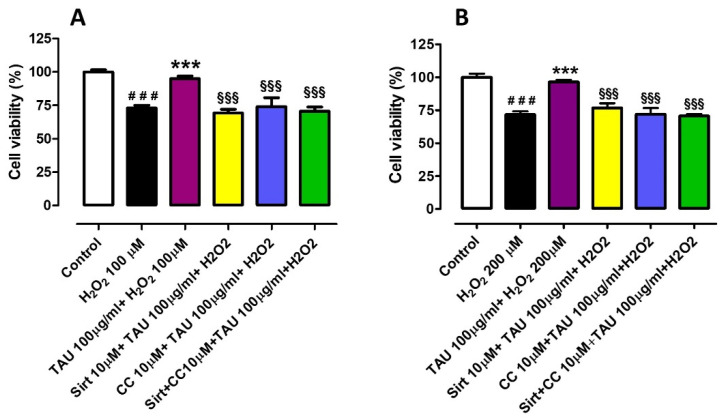
Evaluation of the protective effect induced by Taurisolo^®^ on HUVECs (**A**) and HASMCs (**B**) cell viability, against the H_2_O_2_-induced oxidative damage, in the presence and the absence of selective inhibitors of sirtuins pathway Sirt 10 µM, of AMPK pathway, CC 10 µM, or both. The colored histograms indicate the % of cell viability of treated cells vs. the value of cell viability in cells treated only with vehicle (Control, white histogram). All the experiments were repeated for a minimum of three times and each one was carried out in triplicate (n = 9). The symbols indicate the level of statistical significance calculated by ANOVA one-way, followed by Bonferroni post-test: ^#^ indicates the significance vs. Control, * indicates significance vs. cells treated with H_2_O_2_, § indicates the significance vs. cells treated with Taurisolo^®^ + H_2_O_2_ (^###^ *p* < 0.001; *** *p* < 0.001; ^§§§^ *p* < 0.001).

On the basis of the above results, the concentration of Taurisolo^®^ 100 µg/mL, which demonstrated full protection on both HUVECs and HASMCs, was selected for further investigation on the potential mechanisms of action. On the other hand, on the basis of the composition of the extract rich in resveratrol, a possible involvement of the Sirtuins pathway was evaluated. Resveratrol is reported as an activator of Sirtuin-1 but also of the AMP-dependent protein kinase (AMPK), and these targets show a mutual co-activation. Therefore, the involvement of the AMPK pathway was also evaluated. In order to evaluate the involvement of Sirtuins and AMPK in the protective activity of Taurisolo^®^, the selective blockers of the Sirtuins pathway Sirtinol and the blocker of the AMPK pathway, Compound C (or Dorsomorphin), were administered, both at 10 µM. In this set of experiments, the administration of H_2_O_2_ 100 µM to HUVECs reduced cell viability by about 27% (%cell viability vs. control: 73.1 ± 1.9), and the pre-incubation of Taurisolo^®^ 100 µg/mL induced an almost full preservation of cell viability (%cell viability vs. control: 95.0 ± 1.9). When the administration of Taurisolo^®^ 100 µg/mL was preceded by the pre-incubation of Sirtinol 10 µM, Taurisolo^®^ was unable to protect the endothelial cells against the oxidative damage (%cell viability vs. control: 69.3 ± 2.8). The same inability was observed when Taurisolo^®^ administration was preceded by pre-incubation of Compound C 10 µM (%cell viability vs. control: 74.0 ± 6.7) and also when the two inhibitors, Sirtinol and Compound C, were co-pre-incubated in HUVECs before Taurisolo^®^ administration (%cell viability vs. control: 70.7 ± 3.1) (Figure 2A). The same experimental protocol was applied on HASMCs in which the administration of H_2_O_2_ 200 µM reduced the vascular smooth muscle viability by about 28% (%cell viability vs. control: 71.8 ± 2.4) and the pre-incubation of Taurisolo^®^ 100µg/mL prevented this reduction resulting in a %cell viability vs. control of 96.4 ± 1.6. As already observed in HUVECs, also in HASMCs both the pre-incubation of Sirtinol and the pre-incubation of Compound C inhibited the protective effect exhibited by Taurisolo^®^ (%cell viability vs. control: 76.7 ± 3.6 and 71.8 ± 4.7 respectively), as well as the co-administration of the two inhibitors (%cell viability vs. control: 70.6 ± 1.4) (Figure 2B).

### 3.3. Investigation of the Potential Mechanisms of Action Involved in the Protective Effect Induced by Taurisolo^®^ on HUVECs and HASMCs, against the H_2_O_2_-Induced ROS Production. Evaluation of Sirtuins and AMPK Pathways

**Figure 3:**
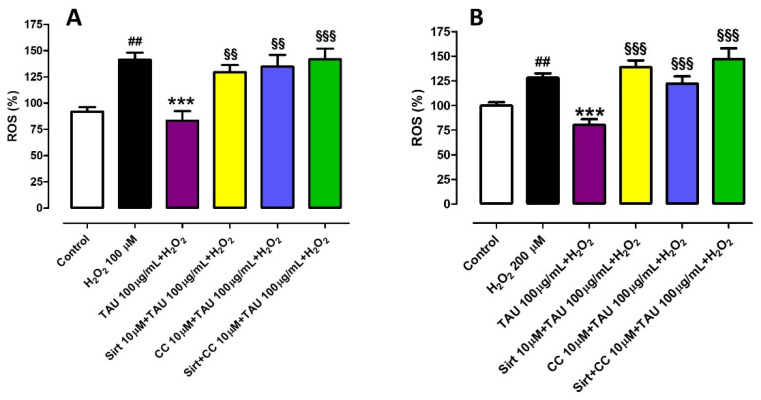
Evaluation of the protective effect induced by Taurisolo^®^ on HUVECs (**A**) and HASMCs (**B**) against the H_2_O_2_-induced ROS-increase, in the presence and in the absence of selective inhibitors of sirtuins pathway Sirt 10 µM, of AMPK pathway, CC 10 µM, or both. The colored histograms indicate the % of ROS levels of treated cells vs. the value of ROS levels in cells treated only with vehicle (Control, white histogram). All the experiments were repeated for a minimum of three times and each one was carried out in triplicate (n = 9). The symbols indicate the level of statistical significance calculated by ANOVA one-way, followed by Bonferroni post-test: ^#^ indicates the significance vs. Control, * indicates significance vs. cells treated with H_2_O_2_, § indicates the significance vs. cells treated with Taurisolo^®^ + H_2_O_2_ (^##^ *p* < 0.01; *** *p* < 0.001; ^§§^ *p* < 0.01; ^§§§^ *p* < 0.001).

Then, the involvement of sirtuins and AMPK pathways was investigated by the measurement of ROS levels. In particular, in HUVEC cells, the administration of H_2_O_2_ induced an increase in ROS level by about 40% (%ROS vs. control: 141.4 ± 6.7) while the pre-incubation of Taurisolo^®^ reduced the ROS level at a level even lower than control (%ROS vs. control: 83.5 ± 9.0). When Sirtinol, Compound C or both were pre-incubated before Taurisolo^®^ and H_2_O_2_, they abolished the preventive effects of Taurisolo^®^ against ROS production (%ROS +Sirtinol vs. control: 129.6 ± 6.7; %ROS +Compound C vs. control: 134.9 ± 11.1; %ROS +Sirtinol + Compound C vs. control: 141.9 ± 10.2) (Figure 3A). On HASMCs, H_2_O_2_ evoked an increase of ROS by about 28% (%ROS vs. control: 128.0 ± 4.6); pre-incubation of Taurisolo^®^ prevented this increase maintaining ROS levels lower than those exhibited in control conditions (%ROS vs. control: 80.3 ± 5.7). In HASMCs, when the administration of Taurisolo^®^ and H_2_O_2_ was preceded by pre-incubation of Sirtinol or Compound C or both, the protective effect exhibited by Taurisolo^®^ against ROS increase was abolished (%ROS +Sirtinol vs. control: 138.8 ± 6.8; %ROS +Compound C vs. control: 122.1 ± 7.1; %ROS +Sirtinol + Compound C vs. control: 147 ± 10.9) (Figure 3B).

### 3.4. Evaluation of the Direct Vasorelaxant Effect Induced by Taurisolo^®^ in Endothelial Intact or Endothelial Denuded Rat Aortic Rings: Involvement of Endogenous NO

**Figure 4:**
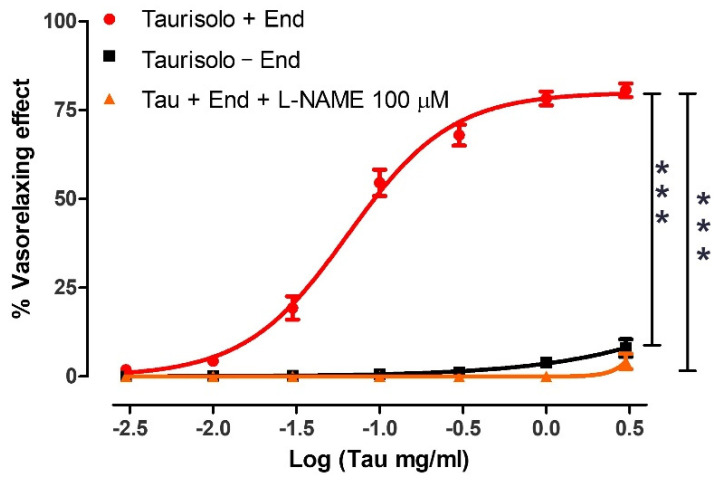
Vasorelaxant effect induced by Taurisolo^®^ on endothelium-intact or endothelium-denuded rat aortic rings. In the red line the concentration-response curve induced by Taurisolo^®^ on endothelium-intact rat aortic rings pre-contracted with KCl 25 mM. In the black line the concentration-response curve evoked by Taurisolo^®^ on endothelium-denuded aortic rings. Finally, in the orange line, the concentration-response curve induced by Taurisolo^®^ on endothelium-intact rat aortic rings in the presence of the inhibitor of the NO biosynthesis, L-NAME 100 μM. The vertical bars indicate the SEM. Six different experiments were performed, each with six replicates (n = 6). The asterisks indicate a significant difference from the red curve obtained on endothelium-intact aortic rings (*** *p* < 0.001).

The administration of increasing concentrations of Taurisolo^®^ to endothelium-intact aortic rings pre-contracted with KCl evoked a clear vasorelaxing effect (Emax = 80.6 ± 1.9 and pEC50 = 1.19 ± 0.03 corresponding about at 0.1 mg/mL) which was completely endothelium-dependent and, in particular, NO-dependent. Indeed, when the administration of Taurisolo^®^ was repeated on endothelium-denuded aortic rings or on endothelium-intact aortic rings pre-treated with the inhibitor of NO-biosynthesis L-NAME, the vasorelaxing effect was completely abolished (Figure 4).

### 3.5. Evaluation of the Direct Vasorelaxant Effect Induced by Taurisolo^®^ in Endothelial Intact or Endothelial Denuded Rat Aortic Rings in the Presence or in the Absence of Specific Inhibitors of Sirtuins (Sirtinol 100 µM) or AMPK (Compound C 100 µM) Pathways

**Figure 5:**
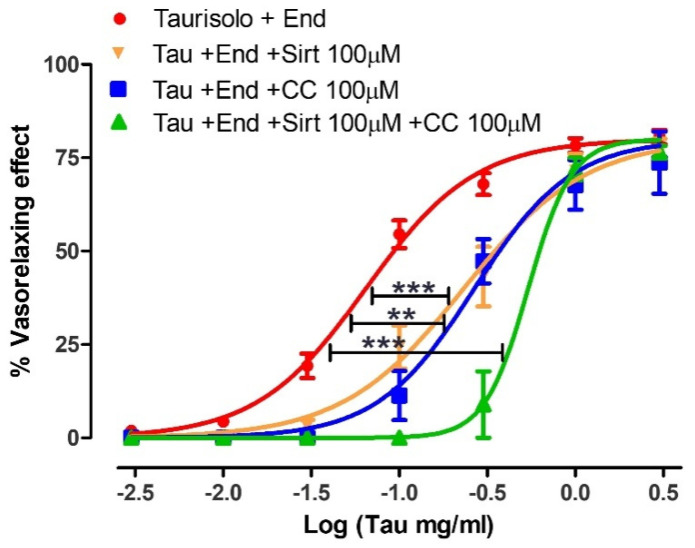
Vasorelaxant effect induced by Taurisolo^®^ on endothelium-intact in the absence or in the presence of Sirtuins and AMPK pathways inhibitors. In the red line the concentration-response curve induced by Taurisolo^®^ on endothelium-intact rat aortic rings, pre-contracted with KCl 25 mM. In the orange line the concentration-response curve evoked by Taurisolo^®^ on endothelium-intact aortic rings pre-treated with the inhibitors of Sirtuins pathway, Sirt 100 µM. In the blue line, the concentration-response curve induced by Taurisolo^®^ on endothelium-intact rat aortic rings in the presence of the inhibitor of the AMPK pathway, CC 100 μM. In the green line, the concentration-response curve induced by Taurisolo^®^ on endothelium-intact rat aortic rings in the presence of both the inhibitors Sirt and CC 100 μM. The vertical bars indicate the SEM. Six different experiments were performed, each with six replicates (n = 6). The asterisks indicate a significant difference from the red curve obtained on endothelium-intact aortic rings (** *p* < 0.01, *** *p* < 0.001).

The vasorelaxant concentration-response curve induced by Taurisolo^®^ was repeated on endothelium-intact rat aortic rings, in the presence of the inhibitor of Sirtuins pathway Sirtinol (100 µM), or the inhibitor of AMPK pathway Compound C (100 µM). In the aortic rings pre-incubated with Sirtinol, similar values of Emax but reduced values of pEC50 were observed (Emax = 76.4 ± 3.8 and pEC50 = 0.63 ± 0.05) if compared to the aortic rings in the absence of inhibitors (red line). The same behavior was observed in aortic rings pre-incubated with Compound C which exhibited similar Emax but reduced pEC50 (Emax = 73.7 ± 3.8 and pEC50 = 0.57 ± 0.06). Finally, when the two inhibitors (Sirtinol and Compound C) were pre-incubated together on endothelium-intact rat aortic rings, they evoked again a similar value of Emax but a further reduction of pEC50 value even if compared with the vasorelaxing curve in the presence of a single inhibitor (Emax = 76.5 ± 1.8 and pEC50 = 0.27 ± 0.04) (Figure 5).

### 3.6. Inhibition of NA-Induced Vasoconstriction by Three Different Concentrations of Taurisolo^®^ Pre-Incubated in Endothelial-Intact Rat Aortic Rings 

**Figure 6:**
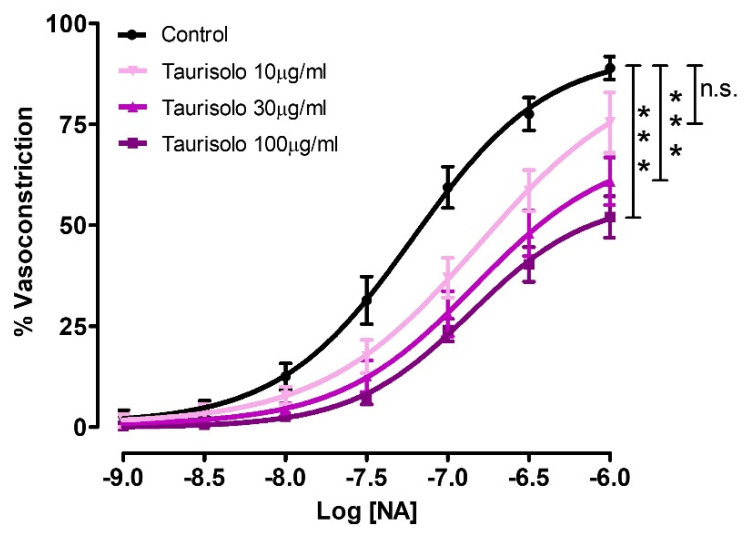
Inhibition of NA--induced vasoconstriction by three different concentrations of Taurisolo^®^. The curves represent the vasocontracturant response to cumulative concentrations of NA in endothelium-intact aortic rings pre-incubated with vehicle (Control) or Taurisolo^®^ at the concentrations of 10, 30 and 100 µg/mL. The vasocontracturant effects are expressed as a% of the contractile responses induced by the administration of KCl 60 mM. The vertical bars indicate the SEM. Six different experiments were performed, each with six replicates (n = 6). The asterisks indicate a significant difference from the Control vasoconstriction curve (line in black) (n.s.= not significant; *** *p* < 0.001).

Besides the direct vasodilating effect, Taurisolo^®^ was also able to inhibit the NA-induced vasoconstriction. In particular, in control endothelium-intact aortic rings, the administration of cumulative concentrations of NA induced a vasoconstricting concentration-response curve reaching an Emax of 88.9 ± 2.9 and a pEC50 of 7.21 ± 0.09. The pre-incubation of endothelium-intact aortic rings with Taurisolo^®^ 10 or 30 or 100 µg/mL, induced a concentration-dependent reduction both of Emax (Emax with Tau 10 µg/mL = 75.4 ± 0.4; Emax with Tau 30 µg/mL = 60.9 ± 5.9; Emax with Tau 100 µg/mL = 52.0 ± 5.1) and of pEC50 values (pEC50 with Tau 10 µg/mL = 6.81 ± 0.20; pEC50 with Tau 30 µg/mL = 6.84 ± 0.18; pEC50 with Tau 100 µg/mL = 6.86 ± 0.11) (Figure 6).

### 3.7. Effect of Taurisolo^®^ In Vivo Chronic Administration on Systolic Blood Pressure Values in SHRs

**Figure 7:**
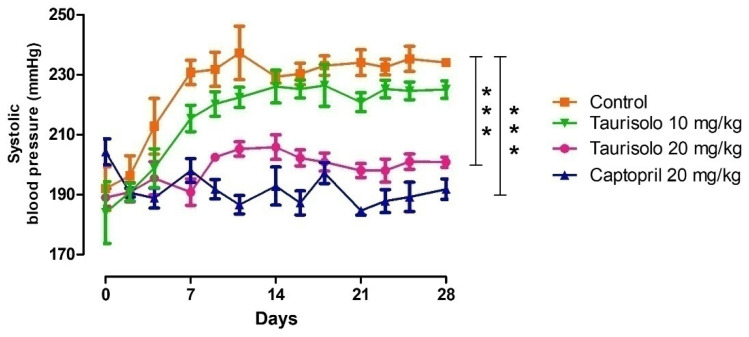
Daily recordings of systolic blood pressure values in SHRs during the in vivo, 4-weeks long chronic, per os treatment with: drinking water (Control group, orange line), Taurisolo^®^ 10 mg/Kg/die (green line), Taurisolo^®^ 20 mg/Kg/die (pink line), Captopril 20 mg/Kg/die (blue line). The vertical bars indicate the SEM. The asterisks indicate a significant difference from the Control SHRs group (*** *p* < 0.001).

As expected, between the 6th and the 10th week of age, SHRs showed a progressive increase in blood pressure. In particular, during the 4 weeks of treatment, the systolic blood pressure of SHRs belonging to the “Control group” increased by about 50 mmHg reaching values of 234 ± 2 mmHg. In SHRs which received Taurisolo^®^ 10 mg/Kg/die the increase of systolic blood pressure was slightly restrained to 225 ± 1 mmHg, while in the SHRs which received Taurisolo^®^ 20 mg/Kg/die the increase of systolic blood pressure values was significantly inhibited, reaching, at the end of the 4th week, values of 200 ± 2 mmHg similar to those recorded in SHRs treated with the reference drug Captopril 20 mg/Kg/die (190 ± 3 mmHg) (Figure 7).

The same recordings were then grouped to express the weekly means of systolic blood pressure values before and during the in vivo, chronic, per os treatment (Table 1).

### 3.8. Effect of Taurisolo^®^ on Glycaemic Levels and the Lipid Panel of the SHRs at the End of the In Vivo Chronic Treatment

At the end of the 4-week-long chronic treatment, the SHRs lipid panel (Total cholesterol, HDL, LDL, and triglycerides) and glycaemic levels (fasting glycemia levels) were recorded as reported in the Methods section. The analysis of these parameters did not reveal significant alterations neither in the lipid panel nor in the fasting blood glucose values of the SHRs belonging to the 4 different groups (data not shown). 

### 3.9. Evaluation of the Protection Induced by Taurisolo^®^ against the Endothelial Dysfunction Exhibited by SHRs

**Figure 8:**
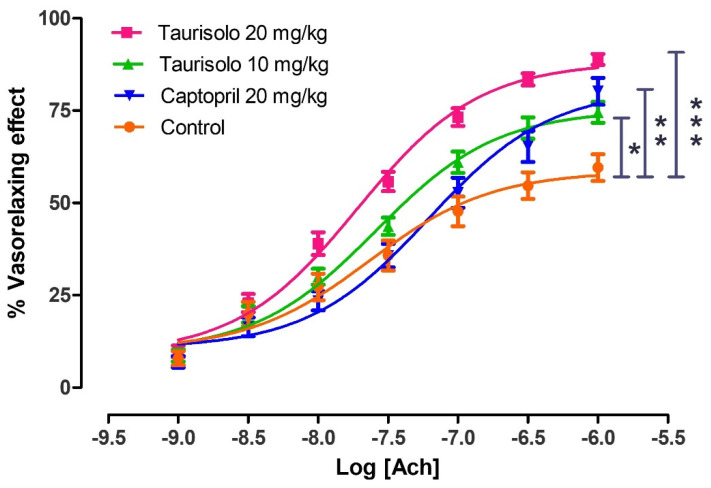
Evaluation of endothelial dysfunction in aortic rings excised by SHRs treated for 4 weeks, orally with: tap water (Control, orange line), Taurisolo^®^ 10 mg/Kg (green line), Taurisolo^®^ 20 mg/Kg (pink line), and the reference drug Captopril 20 mg/Kg (blue line). The experiments were carried out in 5 replicates and the five rings derived from 5 different SHRs (n = 5). The vertical bars indicate the SEM. The asterisks indicate a significant difference from the Control SHRs group (* *p* < 0.05, ** *p* < 0.01, *** *p* < 0.001).

At the end of the 4-week-long chronic treatment, aorta of SHRs was excised and aortic rings were mounted in a bath in order to perform the endothelium functionality test. The aortic rings belonging to SHRs treated only with tap water (Control group) exhibited a reduced vasorelaxing response after Ach administration (Emax = 59.6 ± 3.6), giving evidence of an endothelial dysfunction. The aortic rings from SHRs treated with Taurisolo^®^ 10 mg/Kg/die exhibited a partial but significant recovery of the endothelial function (Emax = 74.5 ± 2.8) similar to that induced by Captopril 20 mg/Kg/die (Emax = 80.2 ± 3.7). Finally, the aortic rings deriving from SHRs treated with Taurisolo^®^ 20 mg/Kg/die exhibited the maximum protection of the endothelial function giving a significant, almost full, vasorelaxing effect (Emax = 88.9 ± 1.5) (Figure 8).

**Figure 9:**
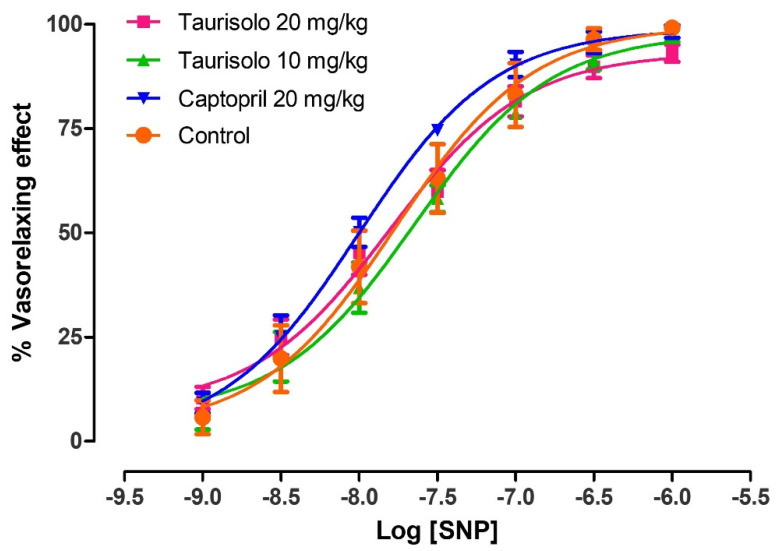
Evaluation of smooth muscle functionality in the aortic rings excised by SHRs treated for 4 weeks, orally with: tap water (Control, orange line), Taurisolo^®^ 10 mg/Kg (green line), Taurisolo^®^ 20 mg/Kg (pink line), and the reference drug Captopril 20 mg/Kg (blue line). The experiments were carried out in 3 replicates and the five rings derived from 5 different SHRs (n = 5). The vertical bars indicate the SEM.

In order to demonstrate that the impaired Ach-induced vasorelaxation observed in control SHRs actually derived from the dysfunction of the endothelial tissue (i.e., reduced biosynthesis of endothelial NO), and was not due to a reduced sensitivity of vascular smooth muscle to NO, the vasorelaxing activity of sodium nitroprusside (SNP) was tested. Indeed, SNP is an NO-donor, that is, a vasorelaxant compound which generates exogenous NO independently from the vascular endothelium. In particular, SNP concentration-response curves were performed on aortic rings deriving from SHRs belonging to the different groups of chronic treatments. The administration of cumulative concentrations of SNP to aortic rings from SHRs treated with tap water (Control), Taurisolo^®^ 10 mg/Kg/die, Taurisolo^®^ 20 mg/Kg/die and Captopril 20 mg/Kg/die evoked almost fully comparable vasorelaxing effects (Figure 9). This confirmed the exclusive role of endothelial dysfunction in the reduced Ach-induced vasorelaxation.

### 3.10. Evaluation of the Protective Effect Induced by Taurisolo^®^ against the Cardiac Hypertrophy Exhibited by SHRs

**Figure 10:**
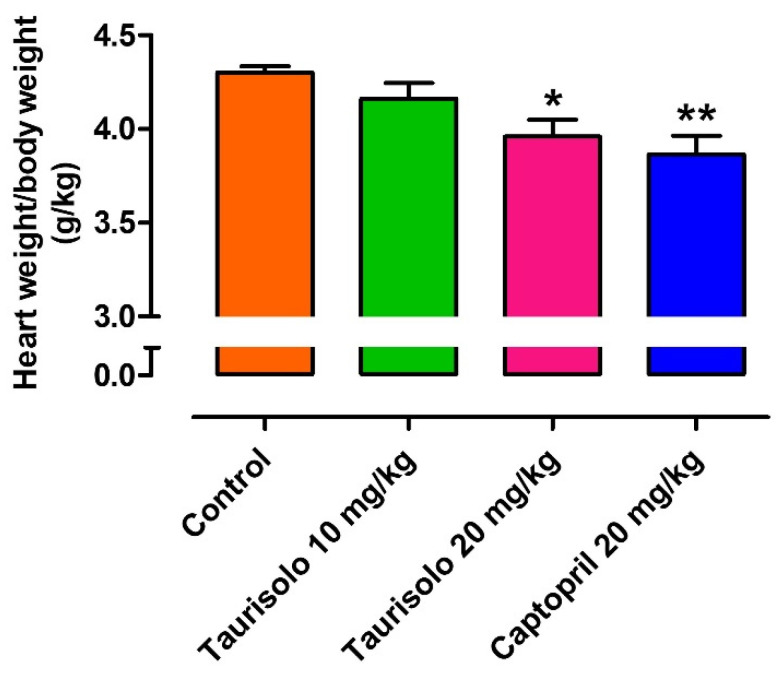
Evaluation of the anti-hypertrophic effect induced by Taurisolo^®^ 10 mg/Kg (green line), Taurisolo^®^ 20 mg/Kg (pink line), and the reference drug Captopril 20 mg/Kg (blue line) on the SHR hearts after 4 weeks of chronic, oral treatment. The experiments were carried out on the hearts from 5 different SHRs for each treatment (n = 5). The vertical bars indicate the SEM. The asterisks indicate a significant difference from the hearts deriving from the Control group (* *p* < 0.05, ** *p* < 0.01).

As the increase of blood pressure values in SHRs is coupled with the development of cardiac hypertrophy, at the end of the 4-week-long treatment the heart of SHRs was excised and weighted. In particular, the reference drug Captopril, as other ACE-inhibitors which are gold standards in the prevention/reversion of cardiac hypertrophy, induced a significant anti-hypertrophic effect if compared to the hearts of the Control group (heart weight/rat weight = 3.87 ± 0.10 g/Kg vs. 4.30 ± 0.03, respectively). Taurisolo^®^ 10 mg/Kg/die induced a slight, non-significant reduction of the cardiac hypertrophy (heart weight/rat weight = 4.16 ± 0.09 g/Kg) while the hearts deriving from SHRs treated with Taurisolo^®^ 20 mg/Kg/die exhibited a value of cardiac hypertrophy significantly different from that exhibited by the hearts excised by SHRs belonging to the control group (heart weight/rat weight = 3.96 ± 0.09 vs. 4.30 ± 0.03) (Figure 10).

### 3.11. Effect of Taurisolo^®^ on Indexes of Coagulation and Clot Retraction

**Figure 11:**
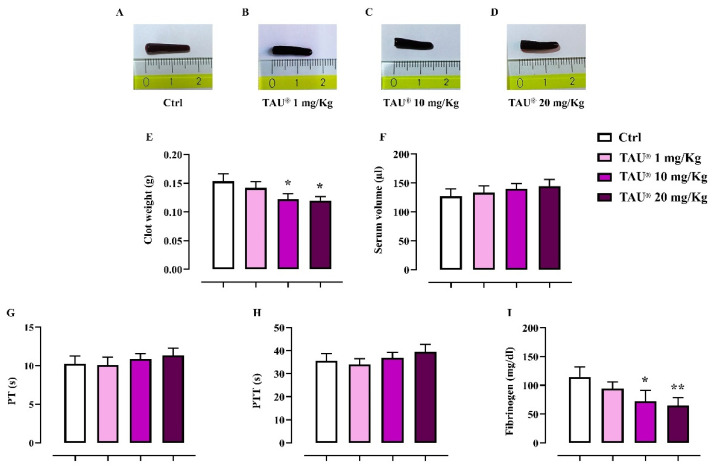
Effect of Taurisolo^®^ on ex vivo clot retraction and coagulation’s indexes (PT, PTT, fibrinogen). The impact of TAU^®^ (1–20 mg/Kg) on platelet activation was evaluated by clots morphology (**A**–**D**), quantification of clot weights (**E**) and residual serum volumes (**F**). The effect on coagulation process and fibrinolytic activity were also determined by measuring PT, PTT and fibrinogen levels (**G**–**I**). Data are presented as means ± SD of n = 6 mice per group. Statistical analysis was conducted by one-way ANOVA followed by Bonferroni’s for multiple comparisons. * *p* ≤ 0.05, ** *p* ≤ 0.01 vs. Ctrl-treated group.

We investigated the indirect effect of Taurisolo^®^ on platelet activation performing a murine ex vivo model of clot retraction. Results were assessed by macroscopic clots morphology (Figure 11A–D) and numerically (clot score) by clots weights (Figure 11E) and residual serum volumes (Figure 11F). It was observed that samples from Taurisolo^®^ -treated mice (10 and 20 mg/Kg; p.o.) were less retracted compared to Control group (*p* ≤ 0.05 vs. Ctrl, Figure 11E), suggesting that Taurisolo^®^ administrated at the higher doses of 10 and 20 mg/Kg decreased the clot retraction rates of platelets. No significant differences were found in the production of serum (Figure 11F). Successively, we analyzed the effect of Taurisolo^®^ on the biochemical indicators of coagulation. As shown in Figure 11I the administration of Taurisolo^®^ at the dose of 10 and 20 mg/kg induced a significant decrease of fibrinogen (expressed as mg/dL) compared to Control group (*p* ≤ 0.05; *p* ≤ 0.01 respectively for Taurisolo^®^ 10 and 20 mg/Kg). No significant differences were found for serum concentrations of prothrombin time and partial thromboplastin time (expressed as seconds) in all experimental groups (Figure 11G,H).

### 3.12. Enrolment and Subject Attrition

**Figure 12:**
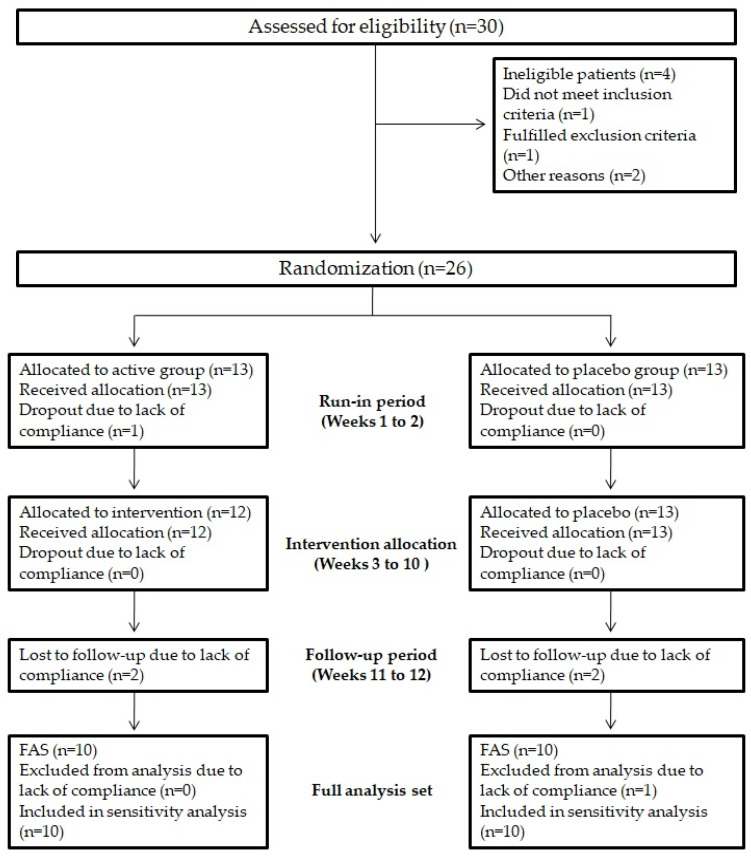
Study flowchart. Study flowchart, according to the consolidated standards of reporting trials (CONSORT). The diagram shows enrolment and primary efficacy endpoints based on patients’ diaries, from prescreening to data collection, and the extent of exclusions, loss to follow-up, and completeness of diary documentation available across the entire trial period. FAS, Full analysis set.

A total of 30 human subjects were screened for eligibility; 4 subjects did not pass the screening phase. Overall, 26 subjects were randomized into active group (n = 13) and placebo group (n = 13). According to CONSORT PRO guidelines [27], Figure 12 shows the flow of study participants through the trial together with the completeness of diary information over the entire treatment period.

### 3.13. Baseline Characteristics of Study Participants

Table 2 reports baseline demographic and clinical characteristics of the 20 subjects completing the study. Overall, the mean age was 24 ± 3 years and BMI 22.3 ± 5 kg/m^2^. The majority of study participants (65%) were non-current smokers and regular physical activity practicers (55%). Serum levels of metabolic parameters were within the normal range for age and sex; serum levels of ox-LDL reflected a strong alteration, while D-ROMs levels fell within the normal range value.

### 3.14. In Vivo Effects of Taurisolo^®^ on Endothelial Function and Oxidative Stress-Related Biomarkers

Clinical characteristic variation before and after 8-week treatment with Taurisolo^®^ or placebo are reported in Table 3.

#### 3.14.1. In Vivo Effects of Taurisolo^®^ on Endothelial Function

**Figure 13:**
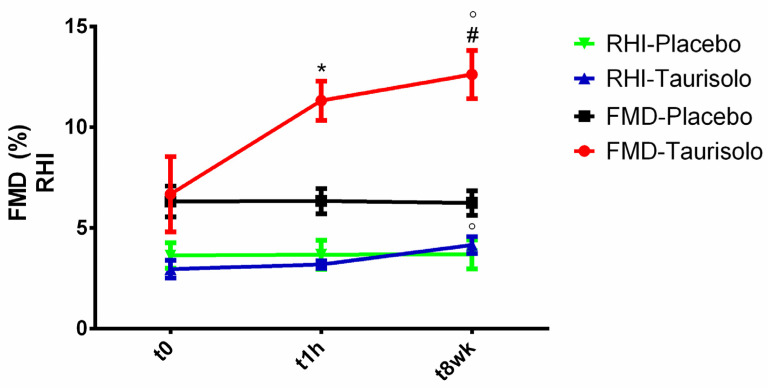
Acute and chronic effect of Taurisolo^®^ on FMD and RHI. The effect of Taurisolo^®^ on endothelial function was evaluated by monitoring FMD and RHI both in acute and after 8-week treatment period. Values are expressed as mean ± SD (three repetitions); statistical significance is calculated by Student’s *t*-test. * *p* < 0.05 t0 vs. t1h; ^#^ *p* < 0.05 t0 vs. t8wk; ° *p* < 0.05 t1h vs. t8wk.

Endothelial function was evaluated at baseline, in the acute phase (1 h after administration of Taurisolo^®^ 800 mg or placebo) and in long-term phase (after 8-week treatment with Taurisolo^®^ 400 mg twice daily or placebo). As shown in Figure 13, we observed significant increases in FMD both in acute (*p* versus baseline = 0.021) and during long-term supplementation (*p* versus baseline = 0.019) in active group. Active group RHI did not show any change in the acute phase (*p* versus baseline = 0.613), whereas a trend toward increase was found after 8-week supplementation of Taurisolo^®^ (*p* versus baseline = 0.079). No significant changes were observed for FMD and RHI in placebo group during the study observation time.

#### 3.14.2. In Vivo Effects of Taurisolo^®^ on Oxidative Stress-Related Biomarkers

**Figure 14:**
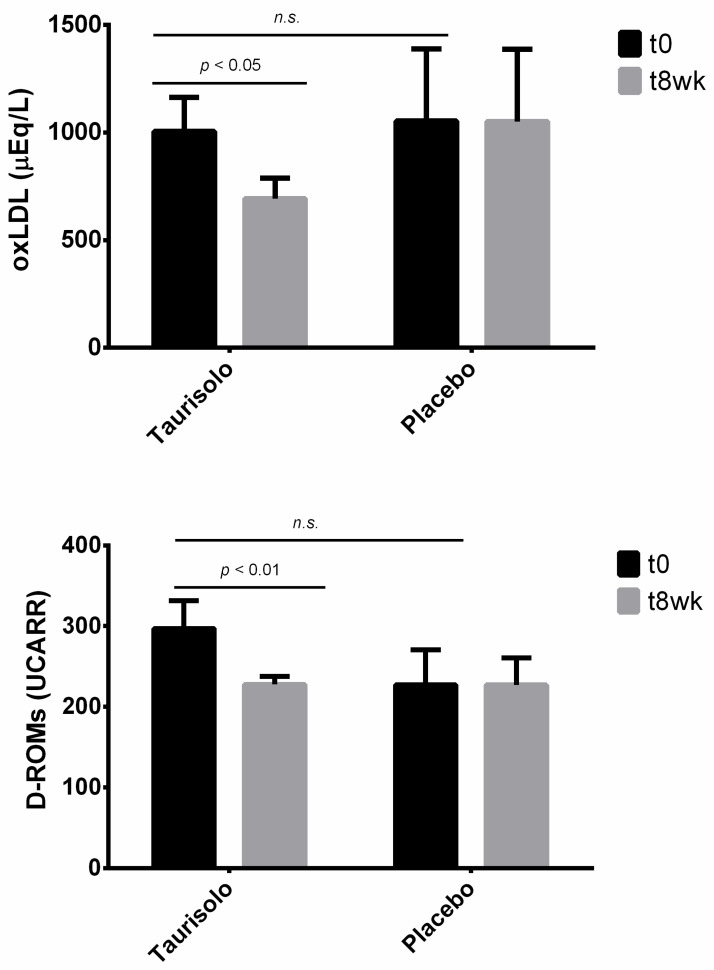
Chronic effect of Taurisolo^®^ on oxidative stress-related biomarkers. Serum levels of oxLDL and D-ROMs, as oxidative stress-related biomarkers, were monitored before and after 8-week treatment with Taurisolo^®^ or placebo. Values are expressed as mean ± SD (three repetitions); statistical significance is calculated by Student’s *t*-test.

Serum levels of oxLDL and D-ROMs were monitored in all study participants before and after 8-week treatment with Taurisolo^®^ (Figure 14). oxLDL and D-ROMs serum levels significantly reduced by 36.36% (*p* = 0.043) and 23.33% (*p* = 0.008), respectively, in active group, while no significant variations were observed in placebo group.

## 4. Discussion

The pharmacological characterization carried out on the cardiovascular properties exhibited by Taurisolo^®^, demonstrated that it represents a nutraceutical product able to induce a protective effect on both endothelium and smooth muscle of the vascular wall, against oxidative stimuli. Such a protection seems to involve two different targets, that is, sirtuins and AMPK, characterized by a mutual co-activation and known to exert a joint action on metabolism, inducing anti-ageing effects and increasing lifespan [28]. Taurisolo^®^ is a grape pomace polyphenolic extract rich in resveratrol and particularly in procyanidins. As reported in scientific literature, resveratrol exerts many of its cardiovascular protective effects, especially against endothelial dysfunction, through the activation of Sirtuin-1 and AMPK [29]. The results obtained on HUVECs and HASMCs confirmed that also Taurisolo^®^ exhibited a protective effect against endothelium and vascular smooth muscle oxidative processes, through the activation of Sirtuins and AMPK pathways. The involvement of the Sirtuins-AMPK pathways in the cardiovascular effects of Taurisolo^®^ seems to be confirmed in the experiments on the rat aortic rings, in which the endothelium-dependent/NO-dependent vasodilation induced by Taurisolo^®^ was impaired when Sirtuins-AMPK pathways were inhibited by the administration of selective blockers. These findings are consistent with the evidence widely described in the literature for resveratrol: activation of Sirtuin-1/AMPK-pathway induces the phosphorylation of eNOS which leads to an increase in NO biosynthesis and to vasodilation [30,31]. Then, the results obtained in in vitro experiments were translated in the in vivo pre-clinical model represented by SHRs, which is a recognized animal model of hypertension, endothelial dysfunction, and cardiac hypertrophy. The sphygmomanometric “tail-cuff” method allows to better reproduce the situation more similar to the clinic one because SHRs are conscious. On the other hand, to avoid the possible alteration of blood pressure due to the animal manipulation, SHRs were trained, for two weeks before the beginning of compound administration, to be handled and to allow blood pressure procedures. As the aim of our work was to evaluate potential prevention of hypertension, endothelial dysfunction, and cardiac hypertrophy by Taurisolo^®^, the SHRs were selected and treated from the 6th to the 10th week of age, since in that interval their blood pressure values, and the consequent degeneration of endothelial and myocardial tissues, are not yet well-established but are in progress. The choice of Captopril as a reference drug was due to its recognized properties to control blood pressure levels and to protect cardiovascular tissues from degenerative processes, especially myocardial remodeling which leads to cardiac hypertrophy. The Taurisolo^®^ doses were selected on the basis of previous studies that demonstrated a protective effect by Taurisolo^®^ against rat brain ischemia-reperfusion injury [32] and to the observation that the doses 10 mg/Kg and 20 mg/Kg in SHRs correspond to about 115 mg and 230 mg/die in humans of about 70 Kg [33]. These doses are well compatible with a daily assumption of this supplement. During the chronic, 4-week long in vivo treatment, oral Taurisolo^®^ 20 mg/Kg/die, demonstrated a significant inhibition of blood pressure increase, from the first week of treatment to the end of the study. As expected, the metabolic parameter represented by fasting blood glucose level and lipidic panel were not altered in SHRs belonging to the Control group and the administration of Taurisolo^®^ did not change significantly the normal metabolic profile of this pre-clinical animal model. Instead, the evaluation of Taurisolo^®^’s impact on endothelial dysfunction highlighted a marked protection of endothelial function by Taurisolo^®^ which reaches the statistical significance at the lowest dosage of 10 mg/Kg/die and which was even better than that exhibited by Captopril at the dosage of 20 mg/Kg/die. Finally, Taurisolo^®^ 20 mg/Kg/die induced also significant prevention of the cardiac hypertrophy developed by SHRs, for which the gold standard is represented by ACE-inhibitors like Captopril.

These evidences were also supported by data from ex vivo clot retraction and coagulation’s indexes. In particular, chronic treatment with Taurisolo^®^ at dose of 10 and 20 mg/kg was able to modify platelet activation and clot formation in mice. Accordingly, fibrinogen levels were down-regulated in Taurisolo^®^-treated mice compared to Ctrl (without any effect on PT and PTT time) suggesting an extrinsic modulation of coagulation’s cascade. 

In order to translate both the in vitro and the animal-based results into the clinical practice, we also investigated the effects of Taurisolo^®^ on endothelial function in humans. We observed that Taurisolo^®^ was able to improve the endothelial function both in acute (1h after oral administration) and chronic (after 8-week administration), as evidenced by increased FMD, accompanied by some changes in RHI (Figure 13). The acute effect may be explained by the high bioavailability of Taurisolo^®^ polyphenols. The acute effects herein observed are in line with previous studies reporting the ability of grape polyphenols to increase FMD within one hour after oral administration [34,35,36,37,38,39,40,41]. Similarly, a number of studies reported the effects of chronic administration of grape polyphenols on endothelial function in terms of increased FMD [42,43,44]. In this case, our results in humans reflect what previously published. According to the available literature, the most relevant mechanism of action for the FMD-increasing effect of chronic polyphenol administration is related to their action on NO production and release that, in turn, is strongly related to FMD. In this sense, our results demonstrating the endothelium-dependent/NO-dependent vasodilation induced by Taurisolo^®^ may serve to provide a solid explanation for our clinic observation, representing a potential mechanism of action for both acute and chronic FMD-increasing effects of Taurisolo^®^.

Since the endothelial function is susceptible to oxidative stress, we also investigated the effects of chronic Taurisolo^®^ administration on oxLDL and D-ROMs, as oxidative stress-related serum biomarkers, observing significant reductions of their circulating levels in the active group compared to placebo (Figure 14). We previously reported the antioxidant potential of Taurisolo^®^ both in human [5] and rats [45]. D-ROMs are useful biomarkers of oxidative stress, determined on the basis of the following ranges: (i) normal: 250–300 UCARR, (ii) border-line: 300–320 UCARR, (iii) low level of oxidative stress: 321–340 UCARR, (iv) middle level of oxidative stress: 341–400 UCARR, (v) high level of oxidative stress: 401–500 UCARR and (vi) very high level of oxidative stress: >500 UCARR, where 1 UCARR = 0.08 mg H_2_O_2_/dL [46,47,48,49]. Baseline D-ROMs levels in this study participants were within the normal range; however, chronic treatment with Taurisolo^®^ significantly reduced the oxidative stress compared to placebo.

In a previous study, we reported the oxLDL-reducing effect of Taurisolo^®^ chronic treatment in human [5]. This is due to the ability of polyphenols to act as hydrogen donors to free radicals, resulting in reduction of biomolecules oxidation, such as LDL [50,51]. Studies also demonstrated that grape polyphenols are effective in the inhibition of copper-catalyzed LDL oxidation [52]. In the present study, serum levels of oxLDL were monitored using the LP-CHOLOX test (Diacron, Grosseto, Italy), aimed to measure the levels of lipid peroxidation-derived hydroperoxides, mainly represented by oxidized cholesterol. According to the manufacturer’s instructions, oxLDL levels are defined: normal (with values ≤599 µEq/L), slightly high (with values ranging from 600 to 799 µEq/L), moderately high (with values ranging from 800 to 999 µEq/L) and very high (with values ≥1000 µEq/L) [53,54]. Our results showed that an 8-week treatment with Taurisolo^®^ significantly reduced serum levels of oxLDL from very high to slightly high levels; on the other hand, no variations were observed in placebo group.

The novelty of these findings consists in the demonstration that the novel extract Taurisolo^®^ exhibited the ability to address an unmet medical need: the protection of the vascular wall and in particular of the endothelial function. These results are original because despite several authors demonstrated similar properties for polyphenols as resveratrol [29], Taurisolo^®^ is not simply a polyphenol; indeed, it contains several substances such as catechin, epicatechin, procyanidins, resveratrol, different flavonoids and organic acids (gallic, syringic, *p*-coumaric, caffeic, ferulic). This heterogeneity makes Taurisolo^®^ an original product for which, to date, no specific study has been performed in order to demonstrate the ability to preserve the vascular endothelium integrity and functionality.

## 5. Conclusions

In conclusion, Taurisolo^®^ emerged as an extract particularly useful for the prevention of that cardiovascular diseases which starts with the alteration of the integrity and the functionality of the vascular wall. These phenomena are mainly based on oxidative processes that characterize the gradual ageing of tissues and which are caused by a fat-rich diet or metabolic disorders like metabolic syndrome, obesity, diabetes, and hypercholesterolemia. This chronic degeneration leads to a dysfunctional endothelium, making it unable to perform its vasodilating and antiplatelet effects mediated by NO and leading to thromboembolic complications. In our work, we aimed to evaluate the unexplored protective effect of Taurisolo^®^ on endothelial tissue and more in general on the vascular function, by also investigating the activation of two targets (Sirtuins and AMPK) mutually connected, as potential mechanism of action. Certainly, as a limitation of our study, we recognize that Taurisolo^®^ is a botanical extract, which contains several substances and this heterogeneity implicates further and different mechanisms of action due to the different molecules. Nevertheless, as reported in discussion [29], we selected sirtuins and AMPK because they were recently described as innovative targets for polyphenols as resveratrol. Of course, we know that sirtuins and AMPK are not the only targets for the vascular mechanism of action for resveratrol [12]. The difficulty to identify one or few mechanisms of action for a botanical extract as Taurisolo^®^ represents one of the most exciting challenge in nutraceuticals studies and surely will be the aim of further investigations. However, the results obtained in this study confirmed the ability of Taurisolo^®^ to arrest the deterioration of the vessels and to protect the endothelial function, inducing the prevention of cardiovascular diseases like hypertension, myocardial infarct, stroke, erectile dysfunction and cognitive impairment. 

## Figures and Tables

**Table 1:** Weekly mean of the systolic blood pressure values (mmHg) recorded in SHRs during the in vivo, chronic, oral treatment with: tap water (Control), Taurisolo^®^ 10 mg/Kg/die, Taurisolo^®^ 20 mg/Kg/die, Captopril 20 mg/Kg/die.

**Week**	**Control**	**Taurisolo^®^ 10 mg/Kg/die**	**Taurisolo^®^ 20 mg/Kg/die**	**Captopril 20 mg/Kg/die**
0	184 ± 6	178 ± 7	185 ± 4	194 ± 2
1	212 ± 4	202 ± 3	192 ± 3 **	193 ± 2 **
2	233 ± 5	223 ± 4	205 ± 2 ***	191 ± 3 ***
3	232 ± 3	224 ± 4	200 ± 2 ***	190 ± 2 ***
4	234 ± 2	225 ± 1	200 ± 2 ***	189 ± 3 ***

** *p* < 0.01, *** *p* < 0.001 indicate the significance levels vs. weekly mean values (mmHg) of the Control SHRs.

**Table 2:** Baseline characteristics of study participants.

Variable	Value ± SD
***Demographic characteristics***	
Subjects (No)	20
Age (years)	24.46 ± 2.99
Male sex (No (%))	11 (55%)
White ethnicity (No (%))	20 (100%)
Smokers (No (%))	7 (35%)
Regular physical activity (No (%))	11 (55%)
***Anthropometric characteristics***	
Weight (Kg)	66.03 ± 11.79
Height (cm)	1.69 ± 0.10
BMI (Kg/m^2^)	22.30 ± 4.87
WC (cm)	76.82 ± 11.52
HC (cm)	98.09 ± 5.37
WHR	0.72 ± 0.25
***Serum parameters***	
Glycaemia (mg/dL)	66.78 ± 12.71
TC (mg/dL)	145.85 ± 32.82
TG (mg/dL)	51.92 ± 16.86
HDL-c (mg/dL)	49.22 ± 12.14
LDL-c (mg/dL)	84.05 ± 34.00
OxLDL (µEq/L)	1031.15 ± 676.50
D-ROMs (UCARR)	259.33 ± 104.94
***Endothelial function***	
FMD (%)	6.48 ± 3.28
***RHI***	*3.26 ± 1.24*

Abbreviations: BMI, body mass index; WC, waist circumference; HC, hip circumference; WHR, waist-to-hip circumference; TC, total cholesterol; TG, triglycerides; HDL-c, high-density lipoprotein cholesterol; LDL-c, low-density lipoprotein cholesterol; oxLDL, oxidised LDL; D-ROMs, reactive oxygen metabolites; FMD, flow-mediated dilation; RHI, reactive hyperemia index.

**Table 3:** Differences between the two intervention groups before and after the 8-week treatment.

Parameters	Taurisolo^®^ (*n* = 10)		Placebo (*n* = 10)		*p*-Value		
	Initial	Final	Initial	Final	Initial (Taurisolo^®^ vs. Placebo)	Taurisolo^®^ (Initial vs. Final)	Placebo (Initial vs. Final)
Age (years)	24.17 ± 0.98	-	24.71 ± 4.11	-	0.757	-	-
Male sex (No (%))	6 (60%)	-	5 (50%)	-	χ^2^ = 0.202; *p* = 0.653	-	-
Smokers (No (%))	4 (40%)	-	3 (30%)	-	χ^2^ = 0.219; *p* = 0.639	-	-
Regular physical activity (No (%))	5 (50%)	-	6 (60%)		χ^2^ = 0.202; *p* = 0.653	-	-
Weight (Kg)	67.53 ± 11.82	67.33 ± 11.23	64.52 ± 12.67	64.35 ± 13.13	0.679	0.672	0.602
Height (cm)	1.67 ± 0.10	-	1.71 ± 0.11	-	0.499	-	-
BMI (Kg/m^2^)	24.24 ± 2.59	24.19 ± 2.42	21.97 ± 2.26	21.89 ± 2.39	0.136	0.738	0.524
WC (cm)	78.75 ± 15.15	78.38 ± 15.19	74.50 ± 5.72	74.64 ± 5.06	0.570	0.188	0.744
HC (cm)	101.92 ± 2.15	101.60 ± 2.54	93.50 ± 4.24	93.28 ± 4.25	**0.002**	0.242	0.189
WHR	0.77 ± 0.14	0.77 ± 0.13	0.80 ± 0.05	0.80 ± 0.05	0.716	0.475	0.403
Glycaemia (mg/dL)	63.32 ± 11.55	74.68 ± 5.82	69.76 ± 13.77	72.66 ± 6.50	0.386	0.234	0.420
TC (mg/dL)	143.67 ± 39.88	154.25 ± 14.48	147.71 ± 28.63	142.57 ± 18.87	0.835	0.055	0.318
TG (mg/dL)	56.83 ± 17.88	52.25 ± 27.28	47.71 ± 16.04	51.57 ± 18.83	0.353	0.474	0.125
HDL-c (mg/dL)	43.63 ± 5.77	53.75 ± 6.55	54.01 ± 14.46	51.56 ± 12.95	0.129	0.062	0.122
LDL-c (mg/dL)	88.67 ± 37.61	90.05 ± 20.73	80.10 ± 33.06	80.70 ± 23.09	0.670	0.744	0.910
oxLDL (µEq/L)	1005.00 ± 389.81	639.50 ± 188.74	1053.57 ± 887.36	1051.43 ± 889.95	0.904	0.043	0.945
D-ROMs (UCARR)	297.08 ± 84.06	227.75 ± 20.01	226.97 ± 116.16	227.11 ± 88.26	0.246	0.008	0.995
FMD (%)	6.67 ± 4.56	12.61 ± 2.92	6.31 ± 2.03	6.24 ± 1.62	0.852	0.019	0.763
RHI	2.95 ± 1.10	4.15 ± 1.03	3.63 ± 1.43	3.69 ± 1.60	0.394	0.079	0.920

No significant differences were evident between the two groups before treatment. Values are expressed as mean ± SD (three repetitions); statistical significance is calculated by Student’s *t*-test: significant *p*-Values are indicated in bold. Abbreviations: BMI, body mass index; WC, waist circumference; HC, hip circumference; WHR, waist-to-hip circumference; TC, total cholesterol; TG, triglycerides; HDL-c, high-density lipoprotein cholesterol; LDL-c, low-density lipoprotein cholesterol; Ox-LDL, oxidised LDL; D-ROMs, reactive oxygen metabolites; FMD, flow-mediated dilation; RHI, reactive hyperemia index.

## Supplementary Materials

The following are available online at https://www.mdpi.com/article/10.3390/nu13051540/s1.
