# Supplementary material for: Vascular Effects of the Polyphenolic Nutraceutical Supplement Taurisolo®: Focus on the Protection of the Endothelial Function
Source: Nutrients. 2021 May 2;13(5):1540. doi: 10.3390/nu13051540 (PMC8147644; doi:10.3390/nu13051540)
Supplement: Supplementary file 1 [file nutrients-13-01540-s001.zip › nutrients-1174954-SI.pdf]

Supplemental material

Taurisolo® supplement

Taurisolo® is a nutraceutical supplement consisting of a polyphenol extract obtained from *Aglianico* cultivar grape, collected during the autumn 2018 harvest. Firstly, the Department of Pharmacy, University of Naples Federico II (Naples, Italy), provided the supplement formulation, then the large-scale production was accomplished by MB-Med Company (Turin, Italy). For the polyphenol extract production, grapes were extracted with water (50 °C), and the solution was filtrated and concentrated and underwent a spray-drying process with maltodextrins as support (5-15%) to obtain a fine microencapsulated powder. The polyphenol profile of Taurisolo® was evaluated by High-Performance Liquid Chromatography-diode array detector (HPLC-DAD, Jasco Inc., Easton, MD, USA) analysis using the method described by Giusti et al., 2017 [8]: Ferulic acid 14.59±0.98 μg/g, Resveratrol 12.55±0.02 μg/g, Caffeic acid 35.00±3.00 μg/g, p-coumaric acid 122.75±2.77 μg/g, Rutin 98.81±7.31 μg/g, Quercetin 135.41±4.69 μg/g, Procyanidin B1 dimer 946.33±55.20 μg/g, Procyanidin B2 dimer 645.89±59.17 μg/g, Syringic acid 310.95±0.01 μg/g, Epicatechin 1696.55±109.60 μg/g, Gallic acid 199.46±4.59 μg/g; Catechin 2499.04±307.41 μg/g.

Cell Cultures

Human aortic smooth muscle cells, HASMCs (Life Technologies, Carlsbad, CA USA), were cultured in Medium 231 (Life Technologies, Carlsbad, CA, USA) supplemented with Smooth Muscle Growth Supplement (Life Technologies, Carlsbad, CA, USA), 1% of 100 units·ml^{-1} penicillin and 100 mg·ml^{-1} streptomycin (Merck KGaA, Darmstadt, Germany). Human umbilical vein endothelial cells, HUVECs (Life Technologies, Carlsbad, CA, USA) were cultured in Medium 131 (Life Technologies, Carlsbad, CA, USA) supplemented with 10% Fetal Bovine Serum, 1% of 100 units·ml^{-1}penicillin and 100 mg·ml^{-1} streptomycin, 1% L-Glutamine, heparin 10U/ml, epidermal growth factor (EGF, 10ng/ml), and basic fibroblast growth factor (bFGF, 5ng/ml) (Merck KGaA, Darmstadt, Germany). Both cell lines were cultured in T75 red cap tissue culture flasks at 37 °C in a humidified atmosphere of 5% CO_2. Cells were split 1:2 once a week and used until passage 20 for HASMCs and 13 for HUVECs.

Evaluation of the cell viability preservation against H_2O_2-induced cell damage in HASMCs and HUVECs

Cells were cultured up to 90% confluence and 24 h before the experiments they were plated into a 96-well cell culture transparent plate (at a density of 10^4 cells per well for HASMCs and 2 × 10^4 cells per well for HUVECs). After 24h, to allow cell attachment, the medium was replaced by fresh culture medium, and cells were treated with Taurisolo® (TAU 10, 30, and 100μg/ml) or vehicle for 1h. After 1h, cells were challenged with the oxidant stimulus, represented by H_2O_2 (200 μM for HASMCs and 100 μM for HUVECs). At the end of the 2h of H_2O_2 incubation, the cell viability was assessed using an aqueous solution of the cell proliferation reagent WST-1 (Roche, Basilea, Switzerland). It was added at a ratio of 1:10 of the total volume of the wells and incubated for 1h at 37 °C in a humidified atmosphere of 5% CO_2, and the absorbance was measured at λ=495 nm by a multiplate reader (EnSpire, Perkin-Elmer, Waltham, MA, USA).

Evaluation of cell viability preservation against H_2O_2-induced cell damage in HASMCs and HUVECs in the presence of Sirtuin and AMPK inhibitors

Cells were cultured up to 90% confluence and 24 h before the experiments, they were plated into a 96-well cell culture transparent plate (at a density of 10^4 cells per well for HASMCs and 2 × 10^4 cells per well for HUVECs). After 24h to allow cell attachment, the medium was replaced and both the cell lines were incubated for 1h with Sirtinol (a sirtuin inhibitor, Tocris Bio-Techne, Minneapolis, MN, USA) or Compound C (also known as dorsomorphin, an AMPK inhibitor, Merck KGaA, Darmstadt, Germany) at the concentration of 10μM, or both, or their vehicle. After 1h of incubation, Taurisolo® (TAU, 100μg/ml) or vehicle (culture medium) was added and incubated for 1h. After 1h, the cells were incubated for2h with the pro-oxidant agent represented by H_2O_2 (200μM for HASMCs, and 100 μM for HUVECs). Cell viability was assessed using an aqueous solution of the cell proliferation reagent WST-1 (Roche, Basilea, Switzerland). It was added at a ratio of 1:10 of the total volume of the wells and incubated for 1h at 37 °C in a humidified atmosphere of 5% CO_2, and then the absorbance was measured at λ=495 nm by a multiplate reader (EnSpire, Perkin-Elmer, Waltham, MA, USA).

Measurement of intracellular H_2O_2-induced ROS production in HUVECs and HASMCs in the presence of Sirtuin and AMPK inhibitors

Cells were treated as reported in the previous paragraph and, at the end of the treatment, intracellular levels of reactive oxygen species (ROS) were measured. In particular, the ROS production was measured using an aqueous solution of the fluorescent probe dihydroethidium (DHE 10μM; Merck KGaA, Darmstadt, Germany) incubated at 37°C for 30 min in the dark and in a humidified atmosphere of 5% CO_2. Fluorescence values corresponding to intracellular ROS production were measured at λex=500nm and λem=580nmby a multiplate reader (EnSpire, Perkin-Elmer, Waltham, MA, USA).

Cell experiments data analysis

The experiments were carried out in triplicate and repeated at least three times (n=9), and the values obtained were expressed as a mean ± standard error (SEM). The data were analyzed by using the ANOVA one way test followed by Bonferroni's Multiple Comparison *post hoc* test; a level of $P < 0.05$ was considered to be a statistical significance limit (*$p< 0.05$; **$p< 0.01$; ***$p< 0.001$).

Animals protocols and ethical statements

All experimental procedures were carried out according to the Code of Ethics of the World Medical Association (Declaration of Helsinki, EU, Directive 2010/63/EU for animal experiments) and following the guidelines of the European Community Council Directive 86-609. The experiments were authorized by the Ethical Committee of the University of Pisa (Protocol number: 0037321/2013) and by the Italian Ministry of Health (authorization number 487/2020-PR). Animal studies were carried out in compliance with the ARRIVE guidelines and the Basel Declaration including the 3Rs concept [9,10]. All procedures were carried out to minimize the number of animals used and their suffering.

Evaluation of the vasorelaxing effect of Taurisolo® on rat aorta rings in the presence and absence of endothelium

3-month-old male Wistar rats (280-350g) housed in cages with free access to food and water, were sacrificed with an overdose of Sodium Thiopental 100 mg/kg (MSD Animal Health, Milan, Italy), and thoracic descending aorta segment was excised, divided into 5mm-rings and set up on 20 mL organ baths, containing Tyrode solution (saline composition: NaCl 136,8; KCl 2,95; $CaCl_2.2H_2O$ 1,80; $MgSO_4.7H_2O$ 1,05; $NaH_2PO_4.H_2O$ 0,41; $NaHCO_3$ 11,9; Glucose 5,5 mM), thermostated at 37°C and saturated with Clioxicarb (95% O_2 and 5% CO_2). Each aortic ring was maintained under a preload of 2 g and, after a period of 30 min of stabilization, KCl 25 mM (CARLO ERBA Reagents S.r.l., Milan, Italy) was added to induce a vasoconstricting effect. After reaching a stable plateau, a test with Acetylcholine (Ach) 10^{-5}M (Merck KGaA, Darmstadt, Germany), was performed to assess the presence of endothelium. As concerns the endothelium-intact aortic rings, a relaxation ≥70% of the KCl-evoked contraction was considered representative of a functional endothelium; on the other hand, the aortic rings exhibiting a relaxation <70% were discarded. As regards the endothelium-removed aortic rings, a relaxation <10% of the KCl-evoked contraction was considered representative of an acceptable lack of the endothelial layer; conversely the rings exhibiting a relaxation ≥10%, were discarded. This procedure was followed by washes to remove Ach and a stabilization period of 20 min [11,12].

At the end of the stabilization period, the aortic rings were challenged again with KCl 25mM to induce vasoconstriction and, once reached a stable plateau, increasing cumulative concentrations of Taurisolo® (0,003; 0,01; 0,03; 0,1; 0,3; 1; 3 mg/mL) were administered in aortic rings with and without endothelium. To verify the involvement of endogenous nitric oxide (NO) in the endothelium-dependent vasorelaxation induced by Taurisolo®, the aortic rings with intact endothelium were pre-incubated with the inhibitor of NO biosynthesis L-NAME 100 μM (Merck KGaA, Darmstadt, Germany) for 20 min, before the administration of increasing cumulative concentrations of Taurisolo®. The vasorelaxing effect promoted by Taurisolo® was expressed as a percentage of the maximum contraction induced by KCl 25 mM. The maximal vasorelaxant efficacy (Emax) represented the maximal vasorelaxing response achieved by the highest concentration of Taurisolo® and it is expressed as a percentage (%) of the contractile tone induced by KCl 25mM. The potency parameter (pEC50) is expressed as the negative logarithm of the molar concentration of Taurisolo® evoking an effect = 50% of

Emax. Both the parameters for efficacy and potency are expressed as mean ± SEM, from aortae of six animals (n = 6) for each different treatment [13,14].

Evaluation of the involvement of Sirtuins and AMPK pathways in the vasorelaxing activity of Taurisolo®

The potential mechanisms of action, accounting for the vasorelaxing effect exhibited by Taurisolo® on the endothelium-intact aortic rings, were investigated through the pre-incubation of the vessels, for 1h, with Sirtinol (a sirtuin inhibitor) 100μM (Tocris Bio-Techne, Minneapolis, MN, USA) or Compound C (also known as dorsomorphin, an AMPK inhibitor) 100μM (Merck KGaA, Darmstadt, Germany) or both at the concentration of 100μM. Therefore, after reaching a stable plateau obtained by administration of KCl 25 mM, increasing concentrations of Taurisolo® (0,003; 0,01; 0,03; 0,1; 0,3; 1; 3 mg/mL) were administered to each aortic ring. The vasorelaxing effect, observed after the administration of each concentration of Taurisolo®, was expressed as a percentage of the maximum contraction induced by KCl 25mM. The maximal vasorelaxant efficacy (Emax) represented the maximal vasorelaxing response achieved with the highest concentration of Taurisolo® and it is expressed as a percentage (%) of the contractile tone induced by KCl 25mM. The potency parameter (pEC50) is expressed as the negative logarithm of the molar concentration of Taurisolo® evoking an effect = 50% of Emax. Both the parameters for efficacy and potency are expressed as mean ± SEM, from aortae of six animals (n = 6) for each different treatment.

Evaluation of the efficacy of Taurisolo® to restrain Noradrenaline (NA)-induced vasoconstriction

Three different concentrations of Taurisolo® (10, 30, 100 μg/mL) or the corresponding vehicle (deionized water) were pre-incubated for 20 min on different isolated endothelium-intact aortic rings. Then, increasing concentrations of Noradrenaline (NA) (Merck KGaA, Darmstadt, Germany) (10^{-9} M; $3x10^{-9}$ M; 10^{-8} M; $3x10^{-8}$ M; 10^{-7} M; $3x10^{-7}$ M; 10^{-6} M) were added in each aortic ring. Finally, after the NA-cumulative concentration–response curve, the aortic rings were washed and, after a stabilization period of 20 minutes, KCl 60 mM was administered to obtain a maximal contraction (100%). The vasocontracting effect of each NA concentration was expressed as a percentage of the vasoconstriction induced by KCl 60 mM. The maximal vasocontracting efficacy (Emax) represented the maximal vasocontracting response achieved with the highest concentration of NA and it is expressed as a percentage (%) of the contractile tone induced by KCl 60mM. The potency parameter (pEC50) is expressed as the negative logarithm of the molar concentration of NA evoking an effect = 50% of Emax. [14].

In vitro experiments statistical analysis

Experimental data were analyzed by a computer fitting procedure (software: GraphPad Prism 6.0). The experiments were conducted in triplicate and repeated at least three times (n=9). The statistical significance was obtained by the Two-Way ANOVA statistical analysis followed by Bonferroni post-test. Values were considered statistically different when $p<0.05$.

Anti-hypertensive effects of Taurisolo® in spontaneously hypertensive rats (SHRs) *in vivo* model

Twenty spontaneously hypertensive rats (SHRs; Charles River Laboratories, Calco, Italy) of 6 weeks of age (starting mean weight: 181 ± 3 g and systolic blood pressure: 186 ± 3 mmHg), were housed in cages with food and water *ad libitum* and were exposed to a 12h dark/light cycle. Although not yet affected by a condition of fully established hypertension, 6-week-old SHR rats were selected because they show a progressive increase in blood pressure up to maximum values around the 10th week of age. After a period of 2 weeks, during which animals were daily conditioned to be handled for measurement of blood pressure according to the"tail-cuff" method, by a BP recorder (BP-2000 Blood Pressure Analysis System, Series II, Visitech System, Apex, NC, USA), they were randomized into 4 groups (5 animals for each group). The 4 groups daily received (*per os*, dissolved in the drinking water) 4 different treatments for 4 weeks: 1) Tap water (control group), 2) Taurisolo® 10mg/kg, 3) Taurisolo® 20 mg/kg, and 4) Captopril 20mg/kg (Merck KGaA, Darmstadt, Germany) used as reference anti-hypertensive drug. Both Taurisolo® and Captopril solutions were daily freshly prepared. During the 4 weeks of treatment, animals were weekly weighed to monitor health status and consider any dose adjustments. Systolic blood pressure was measured three times per week (on alternate days): specifically, conscious animals were placed into containment cages on a 37°C heated platform for 10 min and then systolic

blood pressure values were recorded [15,16]. The statistical significance was obtained by the Two-Way ANOVA statistical analysis followed by Bonferroni post-test. Statistical significance was set at $p<0.05$.

Evaluation of Taurisolo® protection against endothelial dysfunction in SHRs

At the end of the chronic *in vivo* treatment, SHRs were sacrificed by an overdose of Sodium Thiopental (100 mg/kg, i.p.), their thoracic aortas were removed, and rings were set up as previously described, to assess the possible protection of Taurisolo® against endothelial dysfunction. After setting up the aortic rings, they were pre-contracted with NA 1μM and, upon reaching the vasoconstriction plateau, Ach was administered at increasing cumulative concentrations (10^{-9} M; $3x10^{-9}$ M; 10^{-8} M; $3x10^{-8}$ M; 10^{-7} M; $3x10^{-7}$ M; 10^{-6} M) to assess the response of the endothelial component in each treatment group. Some aortic rings were used to verify that vasodilation was due to endothelial deficiency and not to damage of the smooth muscle component. For this purpose, Sodium Nitroprusside (SNP) (CARLO ERBA Reagents S.r.l., Milan, Italy), a NO-donor agent, was administered at the same increasing cumulative concentrations used for Ach [17]. The maximal vasorelaxant efficacy (Emax) represented the maximal vasorelaxing response achieved with the highest concentration of Ach and it is expressed as a percentage (%) of the contractile tone induced by NA 1μM.

The experiments were conducted in fivefold and derived from aortas of 5 different animals (n=5). The statistical significance was obtained by the Two-Way ANOVA statistical analysis followed by Bonferroni post-test. Statistical significance was set at $p<0.05$.

Effect of Taurisolo® on glycemic and lipid parameters in SHRs

At the end of the 4-week of chronic *in vivo* treatment, the lipid (total cholesterol, HDL, LDL, triglycerides) and glycemic profiles were also investigated. After a 18-hour fasting , blood glucose measurement was carried out collecting blood from the tail of conscious animals, while the lipid panel was analyzed from blood collected from the heart of rats previously anesthetized with Sodium Thiopental (100mg/kg, i.p.). The instruments used were Glucocard™ blood glucose meter (Menarini, Florence, Italy) and Cobas b 101 (Roche Diagnostics, Basilea, Switzerland), respectively.

Preventive effects of Taurisolo® against cardiac hypertrophy in SHRs

At the end of the chronic treatment, the heart of each animal was removed, washed, dried, dissected, and finally weighed to assess the preventive effects of Taurisolo® against the development of cardiac hypertrophy in SHRs. Data were expressed as a ratio between heart weight and animal body weight (g/kg) [16]. Significance was obtained with One-Way ANOVA statistical analysis followed by Bonferroni post-test. Data were considered statistically different when $p<0.05$.

Measurement of coagulation factors and fibrinogen

Mice were randomly separated into four experimental groups of six animals of each, balancing body weight variation across groups. Taurisolo® (TAU®) at a dose of 1, 10 and 20 mg/kg and distilled water (TAU vehicle; Ctrl group) were administered orally (p.o.; 200 μl/mouse) for 4 weeks. Thereafter, blood was collected via intracardiac puncture in citrated blood samples to perform haematological investigations of coagulation factors, including prothrombin time (PT; expressed as seconds), partial thromboplastin time (PTT; expressed as seconds) and fibrinogen (expressed as mg/dl) [18]. Blood biochemical examinations were performed by CELL-DYN Sapphire purchased from Abbott SRL (Milan, Italy). Standard laboratory procedures were used for blood sampling and measurements [19] and all procedures were conducted under strictly aseptic conditions.

Clot retraction assay

For clot retraction assay, we adopted the protocol proposed by Law and coll. with slight modifications [20]. Briefly, not-anticoagulated blood samples, obtained by intracardiac puncture (300 μl) were transferred into Microvette® 300 Z (Sarstedt, Verona, Italy) containing clotting activator and incubated at room temperature

for 2h in order to get clots formation. Thereafter, clots were collected and weighed (g), and residual serum volumes (μl) were pipetted as an indirect value of clot reaction [21].

The data and statistical analysis in 2.5 experimental procedures comply with the international recommendations on experimental design and analysis in pharmacology [22] and data sharing and presentation in preclinical pharmacology [23,24]. The results obtained were expressed as the mean ± SD. Statistical analysis were performed by using One-Way ANOVA followed by Bonferroni's for multiple comparisons. GraphPad Prism 8.0 software (San Diego, CA, USA) was used for analysis. Data were considered statistically significant when a value of $p \leq 0.05$ was achieved.

Human clinical studies on the effects of Taurisolo® on endothelial function

Study population and protocol

Study participants were recruited in from February to April 2019 from personnel and students of the Department of Pharmacy. All subjects underwent a standardized physical examination, assessment of medical history (for up to five years before enrolment), laboratory examination, measurement of blood pressure and heart rate, and evaluation of BMI. Body mass index (BMI) was calculated from body height and body weight. Body fat percentage was measured using a body composition analyzer (TBF-310, Tanita Corp., Tokyo, Japan) and systolic blood pressure, diastolic blood pressure, and heart rate, were measured using a HBP-9020 (OMRON COLIN Corp., Tokyo, Japan). At each clinic visit, subjects had to complete three self-administered questionnaires on quality of life aspects, and their diaries were checked for data completeness and quality of documentation to ensure subject comprehension of the diary items. In the frame of a randomized, double-blind, placebo-controlled, parallel-arm clinical trial, subjects aged 22-35 years and with a normal body mass index (BMI from 18.5 and 24.9 kg/m^2) were screened for enrolment. Subjects with any of the following criteria were excluded: BMI < 18.5 kg/m^2 and > 24.9 kg/m^2, diagnosis of cancer, liver disease, renal disease, heart disease, diabetes, hypertension, dyslipidemia, family history of chronic diseases, current pharmacological therapy, current chronic intake of grape polyphenol-based nutraceutical supplements, heavy physical exercise (> 10 h/week), pregnant women, women suspected of being pregnant, women who hoped to become pregnant, breastfeeding, birch pollen allergy, use of vitamin/mineral supplements 2 weeks prior to entry into the study and donation of bloodless than 3 months before the study.

Overall study duration was 12 weeks: 2-week run-in period (without Taurisolo® supplementation); 8-week intervention period with Taurisolo® (400mg twice daily) or placebo (400mg maltodextrin twice daily*)* and 2-week follow-up period. The examinations were performed in an outpatient setting. Clinical examinations and blood sampling were performed after 12h of fasting at weeks 2 and 10. Subjects were instructed to not drink alcohol or perform hard physical activity 2 days prior to blood sampling. Blood samples were collected in the morning and immediately after measurement of heart rate and blood pressure. Blood samples were collected in 10-mL EDTA-coated tubes (Becton–Dickinson, Plymouth, UK) and plasma was isolated by centrifugation (20 min, 2200g, 4 °C). All samples were stored at −80 °C until analysis. All biochemical analyses including fasting plasma glucose, total cholesterol, fasting plasma TG were performed with a Roche Modular Analytics System in the Central Biochemistry Laboratory of our Institution. Low-Density Lipoprotein (LDL) cholesterol and HDL cholesterol were determined by a direct method (homogeneous enzymatic assay for the direct quantitative determination of LDL and HDL cholesterol). During two clinical visits (at weeks 2 and 10), subjects underwent standardized physical examination, assessment of medical history, laboratory examination, measurement of blood pressure and heart rate, and evaluation of BMI. At each clinic visit, patients had to complete three self-administered questionnaires on quality-of-life aspects, and their diaries were checked for data completeness and quality of documentation to ensure patient comprehension of the diary items. Besides clinical visits, standardized telephone interviews were performed every 2 weeks, to verify compliance and increase protocol adherence.

Study participants were randomized into two treatment arms: active group (receiving Taurisolo® treatment) and placebo group (receiving maltodextrins). This study was conducted both in acute and chronic. For the acute study, 800mg of Taurisolo® or maltodextrins were administered to each study participant randomized into the relative intervention group. For the chronic study, subjects in the active group were administered with

400mg Taurisolo® twice daily, while subjects in the placebo group were administered with equivalent dose of maltodextrins.

A total of 30 subjects were enrolled. If a patient dropped out before the intervention period, he/she was replaced by the next eligible patient enrolled at the same centre. Patients, clinicians, core laboratories, and trial staff (data analysts, statisticians) were blind to treatment allocation.

All study participants received oral and written information concerning the study before they gave their written consent. Protocol, letter of intent of volunteers, and synoptic document about the study were submitted to the Scientific Ethics Committee of AO Rummo Hospital (Benevento, Italy). The study was approved by the committee (protocol 123512 of 18/06/2018) and carried out in accordance with the Helsinki declaration of 1964 (as revised in 2000). The subjects were asked to make records in an intake-checking table for the intervention study and side effects in daily reports.

Brachial artery flow-mediated dilation (FMD) and reactive hyperemia index (RHI)

Enrolled patients had to abstain from tobacco, caffeine, and alcohol for at least 12 hours before the examination. All study procedures were performed after overnight fasting in a temperature-controlled room (23°C), after ≥10 min of rest in supine position (a small head pillow was accepted). Brachial artery FMD and RHI were evaluated at baseline (before Taurisolo® or maltodextrin administration), in the acute phase (1 hour after 800 mg Taurisolo® or maltodextrin administration) and at t8 (after 8-weeks of supplementation with 400mg Taurisolo® twice daily or 400mg maltodextrin twice daily). All assessments were performed by the same expert operator, blinded for ongoing treatment. FMD and RHI were measured by ultrasound imaging, as described in the guidelines of the International Brachial Artery Reactivity Task Force [1].

FMD and RHI of the brachial artery were evaluated according to a standardized ultrasound protocol [26] using an automatic edge detection software (Cardiovascular Suite®, FMD studio, QUIPU Srl, Pisa, Italy). Briefly, brachial artery was visualized on the longitudinal plane with ultrasound (10 MHz linear transducer, Esaote®, MyLab 25 Gold, Pisa, Italy). Brachial artery diameter (BAD) and the flow velocity were recorded at rest for 60 seconds and for 240 seconds during reactive hyperemia following 300 seconds of induced ischemia of the forearm (blood pressure cuff placed on the forearm and inflated up to 70 mmHg above the systolic blood pressure). FMD was calculated as (Max post-ischemic BAD – Basal BAD)/Basal BAD x 100. The reactive hyperemia index (RHI) was calculated as (average flow velocity after cuff deflation/flow velocity measured at the baseline) [27]

Blood parameter analyses

All biochemical analyses, including fasting plasma glucose, total cholesterol, fasting plasma triglycerides, high-density lipoprotein (HDL) cholesterol, and low-density lipoprotein (LDL) cholesterol were performed with a fully automated analyzer (Sphera, Edif s.r.l., Rome, Italy).

Serum levels of oxidized low-density lipoproteins (oxLDL) and reactive oxygen metabolites (D-ROMs), as oxidative stress-related biomarkers, were assessed with an automated analyzed (Free Carpe Diem, Diacron International, Grosseto, Italy) using relative commercial kits (Diacron International) according to the manufacture's instruction, as previously reported [6,25].

D-ROMs test (Free Carpe Diem, Diacron International, Grosseto, Italy)

10µl of serum were transferred into 1cm cuvettes containing 1ml of R2 reagent (acetate buffer, pH4.8). The sample-containing mixture was gently mixed and 20µl of R1 reagent (a chromogenic mixture consisting of aromatic alkyl-amine, A-NH2) were added. Cuvettes were mixed by inversion and samples were read at 546nm (5 min, 37°C) on an automated analyzer.

LP-CHOLOX test (Free Carpe Diem, Diacron International, Grosseto, Italy)

10μl of serum were added in a plastic tube containing 1ml of R1 reagent (indicators mixture) and two drops of R2 reagent (reduced iron) were transferred. The mixture was mixed by shaking, incubated at 37°C for 2 min and centrifuged at 1,400 g for 2 min. Supernatants were transferred into 1cm cuvettes and read at 505nm (37°C) on an automated analyzer. Blank was prepared following the same procedure, without the addition of sample.

Data analysis

Unless otherwise stated, all of the experimental results were expressed as mean ± SEM. Statistical analysis of data was performed by the Student's t test or Pearson correlation. The statistic heterogeneity was assessed by using Cochran's test (p<0.1). The I2 statistic was also calculated, and I2>50% was considered as significant heterogeneity across studies. A random-effects model was used if significant heterogeneity was shown among the trials. Otherwise, results were obtained from a fixed-effects model. SD values were calculated from standard errors, 95% CIs, p-values, or t if they were not available directly. Previously defined subgroup analyses were performed to examine the possible sources of heterogeneity within these studies and included health status, study design, type of intervention, duration, total nutraceutical dose, and Jadad score. Treatment effects were analysed using PROC MIXED with treatment and period as fixed factors, subject as random factor and baseline measurements as covariates, and defined as weighted mean difference and 95% CIs calculated for net changes in fecal and serum parameters, and blood pressure values. Data that could not meet the criteria of variance homogeneity (Levenes test) and normal distribution (determined by residual plot examination and Shapiro–Wilks test) even after log transformation were analysed by a nonparametric test (Friedman). The level of significance (α-value) was 95% in all cases ($P < 0.05$).

Analysis set

The full analysis set population included all randomised subjects, and subjects who did not fail to satisfy a major entry criterion. The per protocol set consisted of all subjects who did not substantially deviate from the protocol. This group included subjects for whom no major protocol violations were detected (for example, poor compliance, errors in treatment assignment).

Statistics

All of the experimental data were expressed as mean ± SEM. Statistical analysis of data was carried out by the Student's t test or Pearson correlation. The level of significance (α-value) was 95% in all cases ($P < 0.05$). The degree of linear relationship between two variables was measured using the Pearson product moment correlation coefficient (R). Correlation coefficients (R) were calculated using Microsoft Office Excel.
